# Disruption of Gut Microbiota‐Mediated De Novo NAD^+^ Synthesis Contributes to the Development of Polycystic Ovary Syndrome

**DOI:** 10.1002/advs.202506497

**Published:** 2025-10-13

**Authors:** Ke Chen, Huafeng Geng, Yang Zheng, Hongyang Xie, Rui Qin, Junyang Chen, Cong Ye

**Affiliations:** ^1^ Department of Gynecology China‐Japan Union Hospital of Jilin University Changchun Jilin 130033 China; ^2^ Department of Anesthesiology China‐Japan Union Hospital of Jilin University Changchun Jilin 130033 China

**Keywords:** 3‐HAA, ferroptosis, gut microbiota, NAD^+^, PCOS

## Abstract

Polycystic ovary syndrome (PCOS) is a severe disorder that compromises female ovarian health and elevates the risk of various diseases, including endometrial cancer. The pathogenesis of PCOS remains poorly understood, which has hindered the development of effective interventions. In this study, it is demonstrated that patients with PCOS exhibit significant gut dysbiosis. FMT from PCOS patients (P‐FMT) into mice induced PCOS‐associated symptoms and histological alterations. Notably, both PCOS patients and P‐FMT mice exhibit distinct metabolic profiles in the gut, suggesting a gut microbiota‐mediated metabolic reprogramming. Furthermore, impaired tryptophan metabolism, particularly reduced levels of 3‐hydroxyanthranilic acid (3‐HAA), is observed in both PCOS patients and P‐FMT mice. Administration of 3‐HAA to mice alleviated DHEA‐induced PCOS. Mechanistically, 3‐HAA promoted NAD^+^ synthesis via the de novo biosynthesis pathway, thereby inhibiting DHEA‐induced ferroptosis by modulating the mitochondrial DNA‐cGAS‐STING axis. Collectively, these findings reveal the critical role of gut microbiota‐mediated NAD^+^ synthesis in the pathogenesis of PCOS, underscoring the potential of targeting gut microbiota and NAD^+^ homeostasis as a therapeutic strategy for PCOS prevention and management.

## Graphical abstract

This study demonstrates that patients with PCOS exhibit altered gut microbial profiles, and FMT from PCOS patients induces PCOS‐like symptoms in mice. Specifically, gut dysbiosis reduces 3‐HAA levels in the context of PCOS. Administration of 3‐HAA to mice alleviates PCOS by promoting de novo NAD^+^ synthesis. Furthermore, NAD^+^ attenuates PCOS by inhibiting ferroptosis through limiting the activation of the cGAS‐STING pathway.

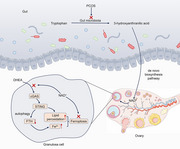

## 1 Introduction

Polycystic ovary syndrome (PCOS) is a prevalent endocrine disorder in humans, characterized by hyperandrogenism, ovulatory dysfunction, polycystic ovarian morphology, and insulin resistance.^[^ ^1^, ^2^ ^]^ PCOS significantly impacts women's reproductive health, leading to reduced ovulation, infertility, and, in severe cases, endometrial hyperplasia, which increases the risk of endometrial cancer.^[^ ^3^ ^]^ To date, the etiology and pathophysiological mechanisms of PCOS remain elusive, hindering the development of effective prevention and treatment strategies. Although studies have indicated that excessive oxidative stress, inflammation in ovarian granulosa cells, and cell death pathways, including ferroptosis, are linked to PCOS,^[^ ^4^, ^5^ ^]^ the precise underlying mechanisms remain to be elucidated.

Recently, studies have demonstrated that the gut microbiota is closely associated with PCOS. In patients with PCOS, distinct gut microbial profiles have been identified, characterized by an enrichment of fecal *Bacteroides* and accompanied by disrupted bile acid metabolism.^[^ ^2^ ^]^ Fecal microbiota transplantation (FMT) from PCOS patients to recipient mice significantly induced PCOS symptoms in mice, including insulin resistance, disrupted estrous cycles, increased numbers of cyst‐like follicles, and fewer corpora lutea.^[^ ^2^ ^]^ These findings were corroborated by another study, which showed that administering antibiotics to deplete the gut microbiota alleviated letrozole‐induced PCOS in mice.^[^ ^6^ ^]^ Regulation of the gut microbiota through butylated starch attenuated PCOS by stimulating the secretion of peptide tyrosine‐tyrosine. A previous study also revealed that administration of *Lactobacillus reuteri* protects mice against circadian dysrhythmia‐induced dyslipidemia via capric acid and GALR1 signaling during PCOS.^[^ ^7^ ^]^ These results highlight the important role of the gut microbiota in PCOS. However, depletion of the gut microbiota did not alleviate dehydroepiandrosterone (DHEA)‐induced PCOS in rats but altered glucolipid metabolism and endocrine functions.^[^ ^8^ ^]^ Moreover, another study identified different gut microbial compositions in PCOS patients, marked by an increase in the fecal *Catenibacterium* and *Kandleria* genera.^[^ ^9^ ^]^ Therefore, further evidence is required to elucidate the role of the gut microbiota in the pathogenesis of PCOS.

Nicotinamide adenine dinucleotide (NAD^+^) is involved in numerous metabolic reactions within cells and plays a critical role in various biological processes, including metabolism, DNA repair, and aging.^[^ ^10^, ^11^ ^]^ NAD^+^ is primarily synthesized via the tryptophan‐mediated de novo biosynthesis pathway and the salvage pathway.^[^ ^11^ ^]^ In the de novo biosynthesis pathway, tryptophan is sequentially metabolized into L‐kynurenine, 3‐hydroxyanthranilic acid (3‐HAA), and quinolinic acid (QA).^[^ ^11^, ^12^ ^]^ QA subsequently serves as a substrate for NAD^+^ synthesis catalyzed by the rate‐limiting enzyme quinolinate phosphoribosyltransferase (QPRT). In the salvage pathway, nicotinamide phosphoribosyltransferase (NAMPT) acts as the rate‐limiting enzyme for NAD^+^ production.^[^ ^12^ ^]^ Notably, both tryptophan metabolism and direct NAD^+^ metabolism are regulated by the gut microbiota.^[^ ^10^ ^]^ Disruption of NAD^+^ metabolism has been linked to several diseases, such as heart failure,^[^ ^13^ ^]^ cancer,^[^ ^14^ ^]^ diabetes,^[^ ^15^ ^]^ and colitis.^[^ ^16^ ^]^ The underlying mechanisms involve the regulation of oxidative stress and inhibition of mitochondrial DNA‐mediated cGAS‐STING activation.^[^ ^12^, ^17^ ^]^ Importantly, impaired NAD^+^ metabolism also contributes to the pathogenesis of PCOS and ovarian senescence.^[^ ^18^, ^19^ ^]^ However, the manner in which the gut microbiota mediates NAD^+^ metabolism during PCOS and its associated mechanisms remains unclear.

Ferroptosis is a regulated form of cell death characterized by excessive lipid peroxidation and intracellular Fe^2+^ accumulation.^[^ ^20^, ^21^ ^]^ Excessive lipid peroxidation is typically associated with elevated oxidative stress and impaired antioxidant capacity in cells. The upregulation of oxidative stress‐related genes, such as PTGS2, and the downregulation of GPX4 are considered classic hallmarks of ferroptosis.^[^ ^20^, ^21^ ^]^ Moreover, the degradation of ferritin heavy chain (FTH) contributes to Fe^2+^ accumulation, which can be modulated through STING‐mediated autophagy.^[^ ^22^, ^23^, ^24^ ^]^ Ferroptosis has been closely linked to various diseases, including cancer, inflammation, and metabolic disorders.^[^ ^20^, ^21^ ^]^ Recent studies have demonstrated that increased ferroptosis plays a role in the pathogenesis of PCOS. Specifically, PCOS patients exhibit elevated levels of malondialdehyde (MDA) and Fe^2+^ in the ovary, along with reduced FTH and GPX4 levels.^[^ ^4^ ^]^ Hyperandrogenism and insulin resistance further promote ferroptosis by reducing GPX4 expression in PCOS rat models.^[^ ^25^ ^]^ Notably, impaired NAD^+^ production facilitates ferroptosis progression. For example, NAD^+^ protects spermatogenesis from ferroptosis via a SIRT2‐dependent mechanism.^[^ ^26^ ^]^ Nicotinamide mononucleotide (NMN) supplementation to enhance NAD^+^ levels alleviates ferroptosis by modulating mitochondrial GPX4 acetylation during acute kidney injury.^[^ ^27^ ^]^ Furthermore, NAD^+^ supplementation inhibits the cGAS‐STING pathway by enhancing mitophagy and restricting mitochondrial DNA release,^[^ ^17^ ^]^ thereby mitigating neuroinflammation and cellular senescence. Additionally, nicotinamide nucleoside treatment reduces STING‐mediated senescence in ataxia telangiectasia,^[^ ^28^ ^]^ suggesting that NAD^+^ may regulate ferroptosis by modulating the cGAS‐STING pathway. However, the precise mechanism underlying NAD^+^‐mediated regulation of ferroptosis in PCOS remains unclear.

In this study, we demonstrated that individuals with PCOS exhibited significant gut dysbiosis characterized by the depletion of commensal *Lactobacillus* and *Alistipes*. FMT from PCOS patients to recipient mice induced PCOS‐like phenotypes. Importantly, both PCOS patients and mice displayed distinct gut microbiota‐mediated metabolic profiles. Disrupted tryptophan metabolism was observed, specifically reduced levels of 3‐HAA and increased levels of 5‐hydroxytryptamine (5‐HT), in both PCOS patients and PCOS mice. Treatment of mice with 3‐HAA alleviated DHEA‐induced PCOS, which was dependent on NAD^+^ production, as evidenced by the fact that inhibition of QAPRT weakened the protective effects of 3‐HAA on PCOS. Mechanistically, NAD^+^ suppressed DHEA‐induced oxidative stress and ferroptosis by inhibiting cGAS‐STING signaling in ovarian granulosa cells. Altogether, our findings suggest that gut microbiota‐mediated de novo synthesis of NAD^+^ contributes to the pathogenesis of PCOS, providing a potential basis for developing gut microbiota‐based interventions for PCOS.

## 2 Results

### 2.1 Patients with PCOS Exhibit Altered Gut Microbiota Profiles Compared to Healthy Individuals

**Figure 1:**
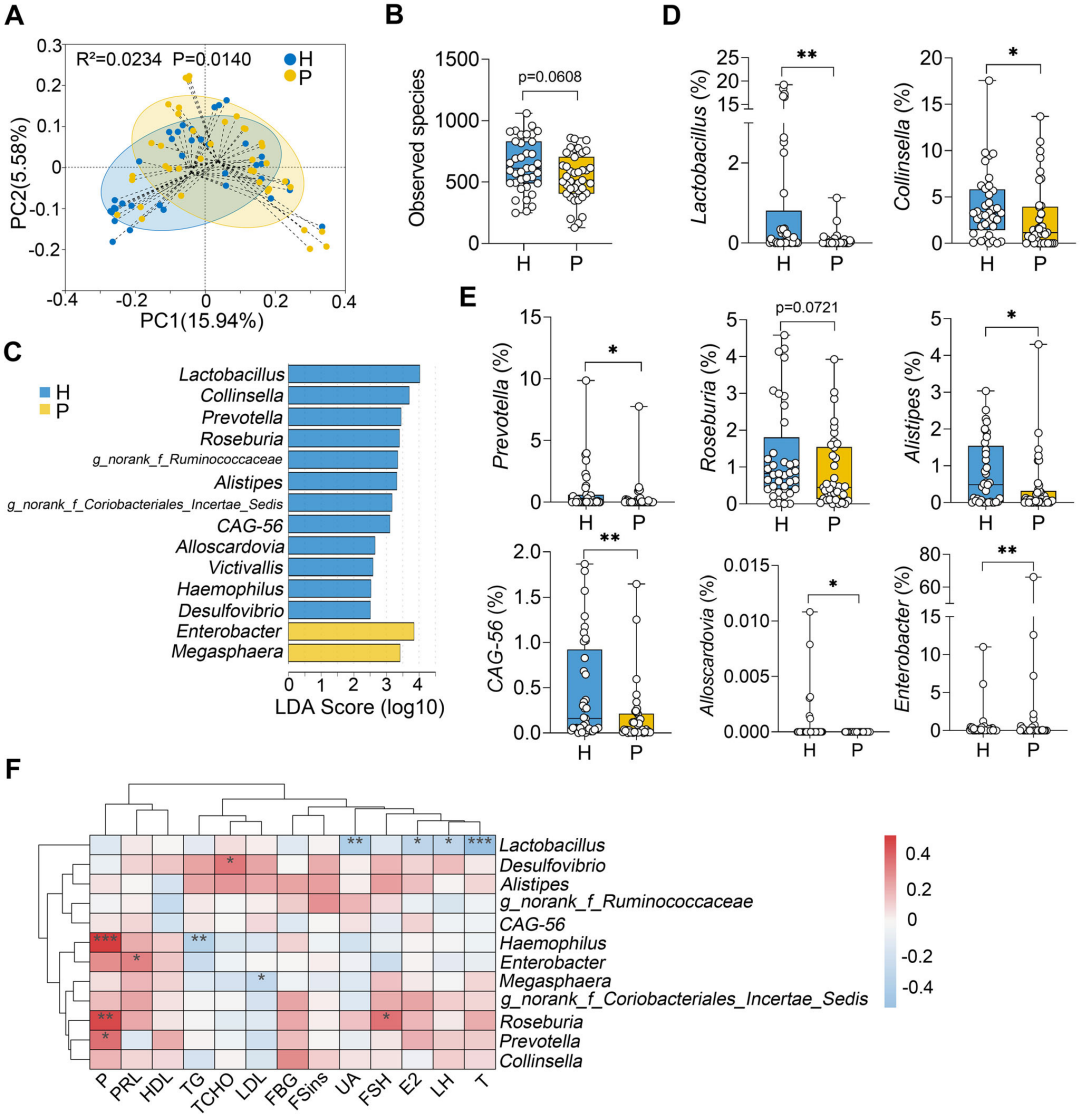
Patients with PCOS exhibit altered gut microbial profiles. A) Gut microbial structure of individuals in the healthy (H) and PCOS (P) groups was analyzed using PCoA score plots based on unweighted UniFrac distance. Adonis was performed for statistical analysis (n = 36–39). B) Observed species richness of the gut microbiota from the H and P groups (n = 36‐39). C) Bacterial taxa commonly enriched in the guts of H and P individuals identified by LEfSe analysis (LDA score (log 10) >2.5). D,E) Relative abundances of differentially abundant genera in the gut microbiota are presented (n = 36–39). F) Correlation analysis between significant gut bacterial taxa and serum parameters was performed using Spearman's correlation. Correlations with an absolute value greater than 0.1 are displayed; red indicates a positive correlation, and green indicates a negative correlation. The intensity of the color reflects the strength of the *Spearman* correlation. Abbreviations: P, Progesterone; PRL, Prolactin; HDL, High‐density lipoprotein; TG, Triacylglycerol; TCHO, Total cholesterol; LDL, Low‐density lipoprotein; FBG, Fasting blood glucose; FSins, Fasting insulin; UA, Uric acid; FSH, Follicle‐stimulating hormone; E2, Estradiol; LH, Luteinizing hormone; T, Testosterone. Data are represented as boxplots (n = 36–39). ^*^ *p* < 0.05 and ^**^ *p* < 0.01 by Mann–Whitney U test (B, D, and E). H, healthy; P, PCOS.

To investigate the gut microbial profiles, 39 patients diagnosed with PCOS according to the 2003 Rotterdam criteria were recruited, along with 36 age‐matched healthy individuals serving as controls. Using 16S rRNA sequencing, principal coordinates analysis (PCoA) based on unweighted UniFrac distance revealed that the gut microbial structure of PCOS patients was distinct from that of the healthy (H) group (**Figure** **1**A). Additionally, a lower observed species richness of the gut microbiota was detected in PCOS patients compared to the healthy group (Figure 1B), which was consistent with reduced alpha diversity indices observed in PCOS patients (Figure S1A, Supporting Information). At the phylum level, an increased abundance of *Proteobacteria* was noted in PCOS patients relative to the H group (Figure S1B, Supporting Information). At the family level, PCOS patients exhibited increased relative abundances of *Lachnospiraceae* and *Enterobacteriaceae*, as well as a decreased abundance of *Ruminococcaceae* (Figure S1C, Supporting Information). At the genus level, distinct gut microbial compositions were identified in PCOS patients compared to healthy individuals (Figure S1D, Supporting Information). To further identify differentially abundant bacterial taxa, linear discriminant analysis effect size (LEfSe) was performed, revealing that 12 genera were depleted in PCOS patients, including *Lactobacillus*, *Collinsella*, *Prevotella*, *Roseburia*, and *Alistipes*, while *Enterobacter* and *Megasphaera* were enriched (Figure 1C–E; Figure S1E, Supporting Information). Moreover, *Spearman* correlation analyses demonstrated that the depletion of *Lactobacillus* in the PCOS group was negatively correlated with serum testosterone (T), luteinizing hormone (LH), estradiol (E2), and uric acid (UA) levels (Figure 1F). Collectively, these findings indicate that PCOS patients exhibit significant gut dysbiosis.

### 2.2 FMT from Patients with PCOS into Mice Induces the Development of PCOS‐Like Phenotypes in Mice

**Figure 2:**
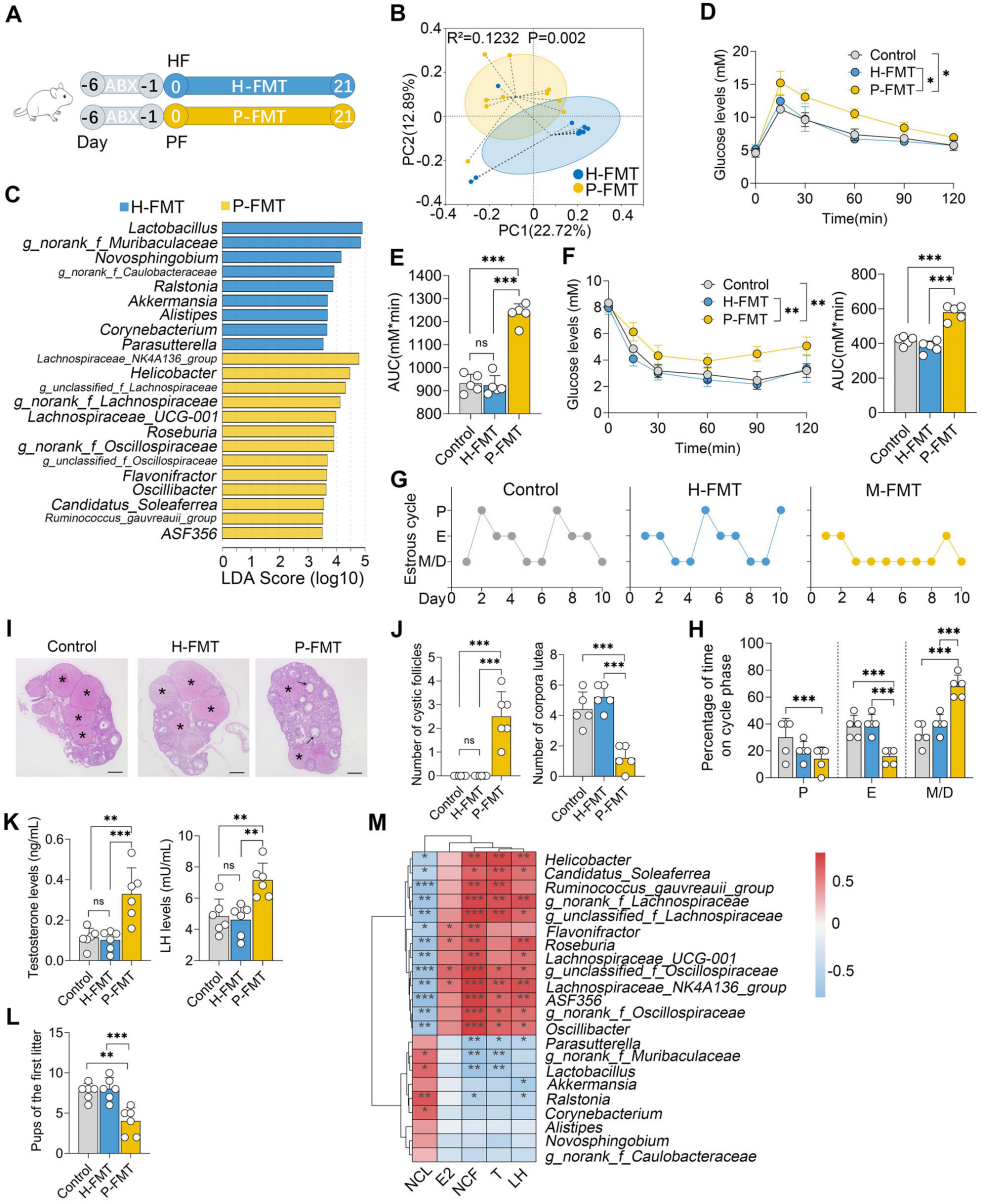
FMT from patients with PCOS to mice induces PCOS‐like symptoms in mice. A) Schematic diagram of FMT. Mice were orally administered a cocktail of antibiotics (ABX, including 200 mg kg^−1^ ampicillin, neomycin, metronidazole, and 100 mg kg^−1^ vancomycin) daily for five consecutive days to deplete the indigenous gut microbiota. After replacing ABX with water for one day, these mice were inoculated with prepared gut microbial suspensions derived from healthy donors (H‐FMT) or PCOS patients (P‐FMT). B) Gut microbial structure of mice in the H‐FMT and P‐FMT groups was analyzed using PCoA score plots based on unweighted UniFrac distance. Adonis was performed for statistical analysis (n = 10). C) Differentially enriched bacterial taxa in the guts of H‐FMT and P‐FMT mice were identified using LEfSe analysis (LDA score (log 10) >3.5). D,E) Glucose tolerance test (GTT) results demonstrated altered glucose metabolism in different FMT‐treated mice (n = 5). F) Insulin tolerance test (ITT) revealed that P‐FMT mice exhibited higher serum glucose levels compared to Control and H‐FMT groups (n = 5). G,H) Estrous cycle determination via vaginal smears indicated disrupted cycles in P‐FMT mice. Stages included proestrus (P), estrus (E), metestrus (M), and diestrus (D). I) Representative hematoxylin and eosin (H&E)‐stained images of ovarian tissues from Control, H‐FMT, and P‐FMT groups (scale bar, 200 µm). J) Quantitative analysis of cystic follicles (NCF) and corpora lutea (NCL) based on H&E‐stained sections (n = 5). K) Serum testosterone and LH levels in the indicated mice (n = 6). L) Number of pups in the first litter from mice subjected to FMT (n = 6). M) *Spearman* correlation analysis revealing the correlation between significant gut bacterial taxa and PCOS‐associated parameters. Correlations with an absolute value greater than 0.1 are shown; red indicates a positive correlation, and green indicates a negative correlation. The intensity of the color reflects the strength of the Spearman correlation. Data are presented as mean ± SD. Each dot represents an individual mouse (B, D–H, J, and K, n = 5‐10). ^*^ *p* < 0.05, ^**^ *p* < 0.01, and ^***^ *p* < 0.001 by one‐way ANOVA followed by Tukey's post hoc test (E, F, H, J, K, and L) or two‐way ANOVA (D and F).

To investigate the role of gut microbiota in the pathogenesis of PCOS, FMT was conducted using samples from healthy individuals and those with PCOS in mice (**Figure** **2**A). A Venn diagram revealed that 140 bacterial species were shared between donor and recipient mice (Figure S2A, Supporting Information). PCoA core plots further demonstrated that the gut microbial structures were distinct among different donors and recipient mice (Figure S2B, Supporting Information). Subsequently, we observed that mice receiving FMT from individuals with PCOS (P‐FMT) exhibited a markedly different gut microbial structure compared to those transplanted with healthy fecal microbiota (H‐FMT) (Figure 2B; Figure S2C, Supporting Information). At both the phylum and genus levels, the gut microbial compositions in P‐FMT and H‐FMT mice showed significant differences but closely resembled the profiles of their respective donors (Figure S2D,E, Supporting Information), confirming the success of FMT. LEfSe analysis indicated that, consistent with the donor profiles, *Lactobacillus* and *Alistipes* were depleted in P‐FMT mice relative to H‐FMT mice (Figure 2C; Figure S2F, Supporting Information). Furthermore, mice transplanted with fecal microbiota from individuals with PCOS exhibited increased insulin resistance compared to those subjected to FMT from healthy donors, as evidenced by glucose tolerance tests (GTTs) and insulin tolerance tests (ITTs) (Figure 2D–F). Additionally, P‐FMT mice displayed a disrupted estrous cycle compared to those in the H‐FMT group (Figure 2G,H). Histological analysis revealed that mice subjected to P‐FMT exhibited an increased number of cyst‐like follicles and a decreased number of corpora lutea compared with those in the H‐FMT group, which displayed normal ovarian profiles characterized by follicles at various stages of development and appropriate numbers of corpora lutea (Figure 2I,J). Additionally, mice subjected to P‐FMT exhibited significantly higher levels of testosterone and luteinizing hormone but not estradiol compared with those in the H‐FMT group (Figure 2K; Figure S2G, Supporting Information). Fertility tests demonstrated that mice in the P‐FMT group produced fewer pups in their first litters after mating compared with those in the H‐FMT group (Figure 2L). *Spearman* correlation analysis indicated that *Lactobacillus* depletion in the P‐FMT group was negatively correlated with serum testosterone levels and the number of cystic follicles, while positively correlated with the number of corpora lutea (Figure 2M). Collectively, these findings suggest that gut dysbiosis is closely associated with the pathogenesis of PCOS.

### 2.3 The Disrupted Kynurenine Pathway of Tryptophan Metabolism is Associated with Gut Microbiota‐Mediated Pathogenesis in PCOS

**Figure 3:**
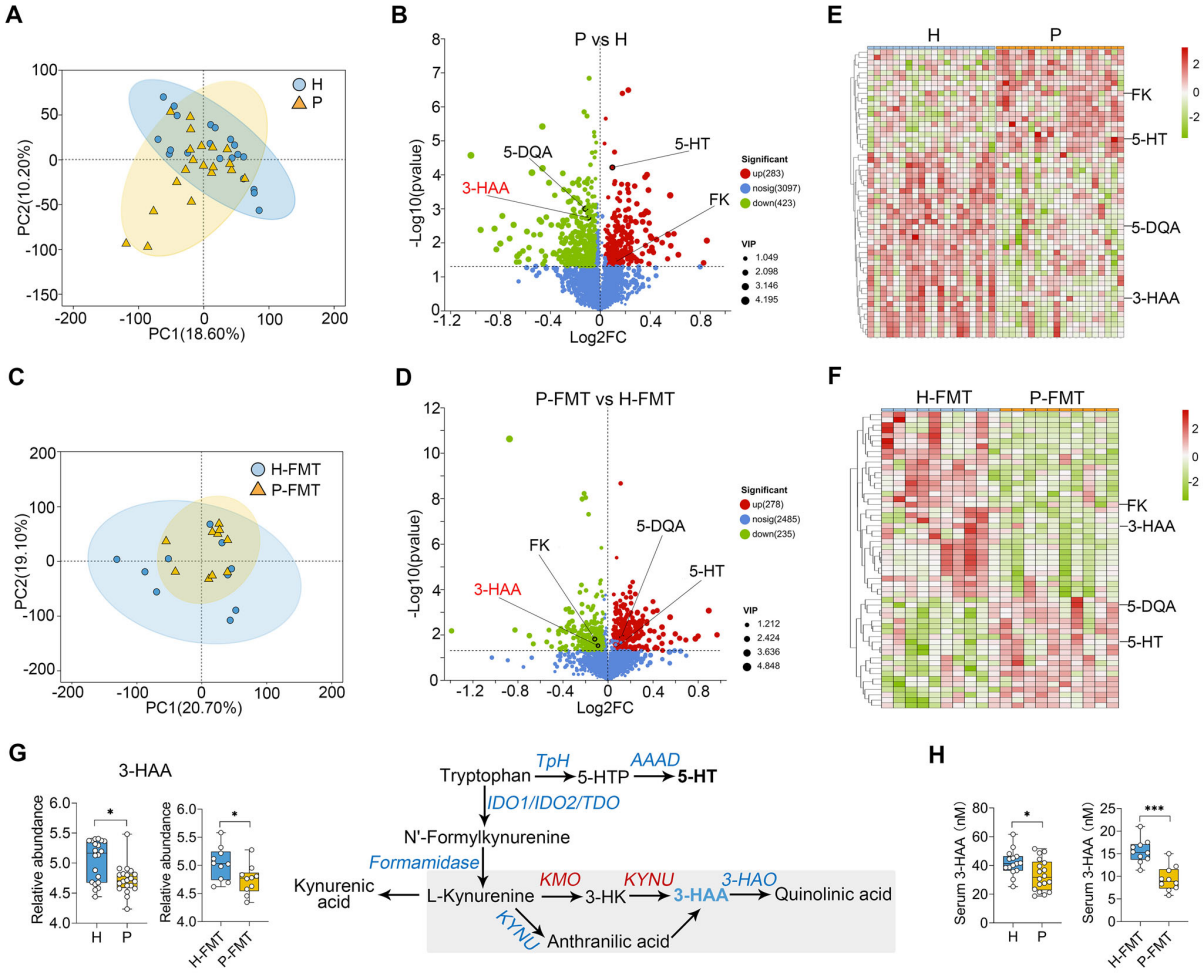
The disrupted kynurenine pathway of tryptophan metabolism was associated with gut microbiota‐mediated pathogenesis in PCOS. A) PCA score plots revealed distinct metabolic profiles between the H and P groups (n = 20). B) Volcano plots identified 283 up‐regulated and 423 down‐regulated differential metabolites in fecal samples from individuals with PCOS compared to those in the H group. C) PCA score plots demonstrated distinct metabolic characteristics between the H‐FMT and P‐FMT groups (n = 10). D) Volcano plots highlighted 278 up‐regulated and 235 down‐regulated differential metabolites in the P‐FMT group relative to the H group. E,F) A heatmap illustrated shared differential metabolites between donor and recipient mice, with metabolites related to tryptophan metabolism, including FK (N'‐formylkynurenine), 5‐HT (5‐hydroxytryptamine), 5‐DQA (5‐dehydroquinic acid), and 3‐HAA (3‐hydroxyanthranilic acid), being marked. G) The relative abundances of 3‐HAA in humans and recipient mice, along with their metabolic pathways, were presented. 5‐HT is synthesized from tryptophan via *TpH* (tryptophan hydroxylase) and *AAAD* (aromatic amino acid decarboxylase). *IDO* (indoleamine 2,3‐dioxygenase) 1/2/*TDO* (tryptophan 2,3‐dioxygenase) catalyze the conversion of tryptophan to FK and L‐Kynurenine, which are further metabolized into 3‐HAA by *KMO* (kynurenine 3‐monooxygenase) and *KYNU* (kynureninase). Metabolites are indicated in black, enzymes in blue, and enzymes encoded by both host and microbes in red. H) Serum 3‐HAA levels in humans and recipient mice. Data are expressed as boxplots (n = 10–20). ^*^ *p* < 0.05 and ^***^ *p* < 0.001 by Mann–Whitney *U* test (G and H).

Gut microbiota‐mediated metabolic changes have been reported to play a significant role in many diseases.^[^ ^29^, ^30^ ^]^ To investigate the gut microbiota metabolism in the pathogenesis of PCOS, we employed untargeted metabolomics. The primary metabolites and enriched pathways were identified using the Human Metabolome Database (HMDB) and the Kyoto Encyclopedia of Genes and Genomes (KEGG) (Figure S3A,B, Supporting Information). Principal component analysis (PCA) and partial least squares discriminant analysis (PLS‐DA) score plots of metabolites revealed that individuals with PCOS exhibited significant separation from healthy controls (**Figure** **3**A; Figure S3C, Supporting Information). A total of 706 significantly different metabolites were identified in fecal samples from PCOS individuals compared to those from the H group, including 423 down‐regulated and 283 up‐regulated metabolites (Figure 3B). To confirm the alterations in metabolites regulated by the gut microbiota, we conducted metabolomics analysis in FMT mice. As expected, mice subjected to P‐FMT showed dramatic changes in their fecal metabolic profiles compared to C‐FMT mice (Figure 3C; Figure S3D–F, Supporting Information). Specifically, 513 different metabolites were identified in P‐FMT mice relative to C‐FMT mice, comprising 235 down‐regulated and 278 up‐regulated metabolites (Figure 3D). Notably, 56 metabolites were commonly altered in both humans and FMT mice (Figure 3E,F). Among these, 37 metabolites exhibited consistent changes between humans and FMT mice, with 12 being up‐regulated and 25 being down‐regulated in both PCOS individuals and P‐FMT mice compared to healthy individuals and H‐FMT mice, respectively (Figure 3E,F). Consistent with the KEGG‐enriched pathway analysis (Figure S3B,E, Supporting Information), several metabolites, including N″‐formylkynurenine (FK), 3‐HAA, 5‐dehydroquinic acid, and serotonin (also known as 5‐hydroxytryptamine, 5‐HT), were associated with tryptophan (Trp) metabolism (Figure 3E,F). N″‐formylkynurenine, 3‐HAA, and 5‐dehydroquinic acid are intermediates in the kynurenine pathway (KP) of Trp metabolism (Figure 3G). Specifically, N′‐formylkynurenine is generated from Trp through the action of indoleamine 2,3‐dioxygenase (*IDO*) 1/2 or tryptophan 2,3‐dioxygenase (*TDO*) in both immune and epithelial cells.^[^ ^12^, ^31^ ^]^ It is subsequently metabolized into 3‐hydroxykynurenine (3‐HK) by various enzymes and further converted into 3‐HAA via kynureninase (*KYNU*) or kynurenine 3‐monooxygenase (*KMO*).^[^ ^32^ ^]^ 3‐HAA can be metabolized into quinolinic acid (QA) and serves as a substrate for the de novo synthesis of nicotinamide adenine dinucleotide (NAD^+^) in the host^[^ ^32^ ^]^ (Figure 3G). 5‐HT is primarily produced by enterochromaffin cells via tryptophan hydroxylase 1 (*TpH1*). However, only 3‐HAA and 5‐HT exhibited consistent changes between donor and recipient mice (Figure 3E–G; Figure S3G, Supporting Information), which aligns with prior findings that *KYNU* and *KMO* in the KP pathway may be microbially encoded.^[^ ^33^ ^]^ Similarly, we further confirmed that serum 3‐HAA levels were decreased in PCOS patients and P‐FMT mice compared with healthy individuals and H‐FMT mice, respectively (Figure 3H). Notably, since 3‐HAA levels were reduced in both PCOS individuals and P‐FMT mice, we hypothesize that 3‐HAA exerts a protective role in PCOS pathogenesis. Collectively, these findings suggest that dysregulation of the KP is associated with gut microbiota‐mediated mechanisms underlying PCOS development.

### 2.4 Administration of 3‐HAA Alleviates PCOS in Mice

**Figure 4:**
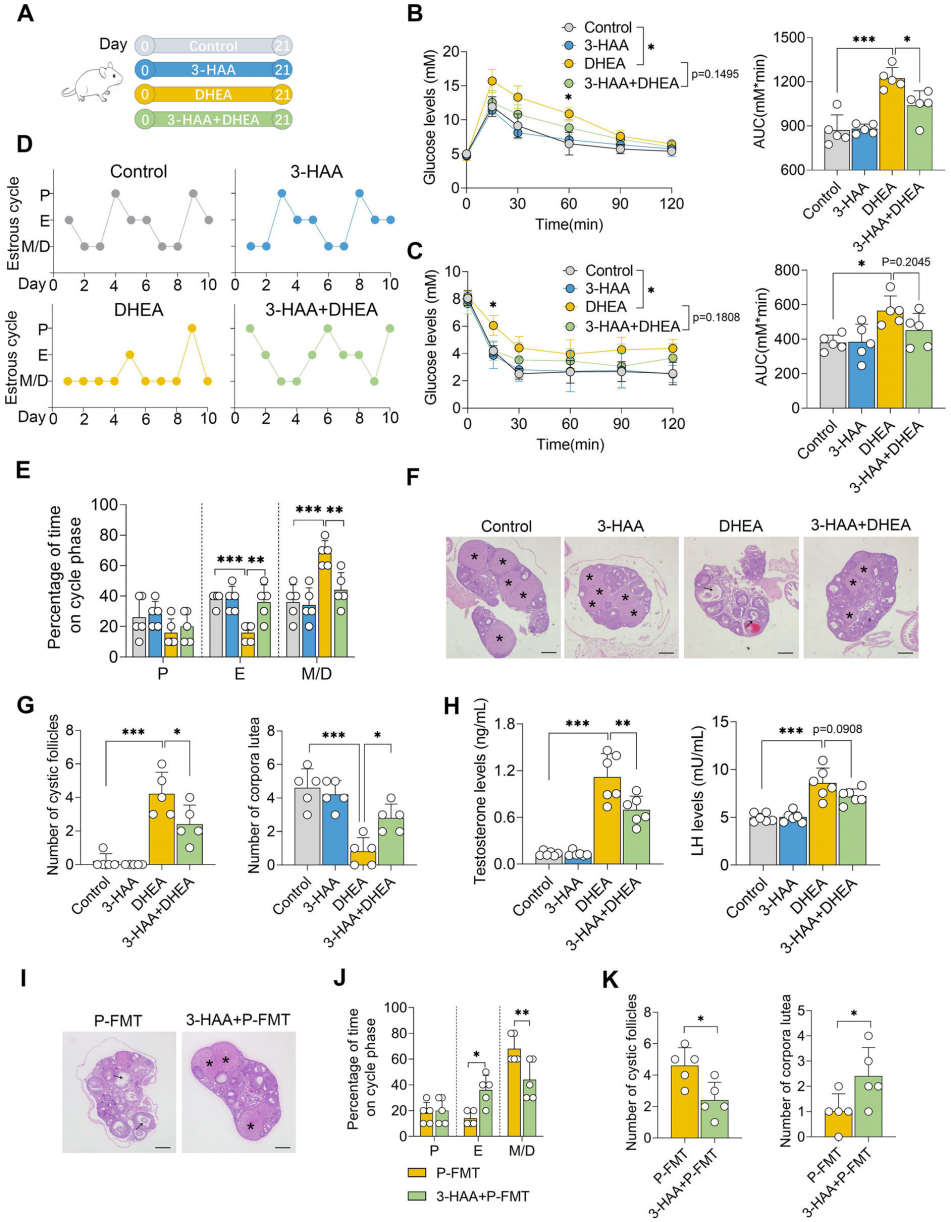
Administration of 3‐HAA alleviates PCOS in mice. A) Schematic diagram of 3‐HAA treatment in DHEA‐induced PCOS mice. Female mice at the prepubertal stage (21‐day‐old) were intraperitoneally administered with 200 mg kg^−1^ 3‐HAA daily and subcutaneously injected with DHEA (60 mg kg^−1^) daily for 21 days. B) GTT assay results of different treated groups (n = 5). Statistical analysis was performed using two‐way ANOVA. ^*^ *p* < 0.05 compared to the DHEA group. C) ITT assay demonstrated that 3‐HAA treatment mitigated DHEA‐induced insulin resistance (n = 5). ^*^ *p* < 0.05 compared to the DHEA group. D,E) Estrous cycle determination based on vaginal smears. P, proestrus; E, estrus; M, metestrus; D, diestrus. F) Representative H&E‐stained images of ovarian tissues from indicated groups (scale bar, 200 µm). G) Quantitative analysis of cystic follicles and corpora lutea based on H&E‐stained sections (n = 5). H) Serum testosterone and LH levels in indicated groups (n = 6). I–K) P‐FMT mice were treated with 3‐HAA for 21 days. (I) Representative H&E‐stained images of ovarian tissues from the indicated groups (scale bar, 200 µm). (J) Estrous cycle determination based on vaginal smears. (K) Quantitative analysis of cystic follicles and corpora lutea based on H&E‐stained sections (n = 5). Data are presented as mean ± SD. Each dot represents an individual mouse (B, C, E, G, H, J, and K, n = 5–6). ^*^ *p* < 0.05, ^**^ *p* < 0.01, and ^***^ *p* < 0.001 by one‐way ANOVA followed by Tukey's post hoc test (E, G, H, J, and K) or two‐way ANOVA (B and C).

To further elucidate the role of 3‐HAA in PCOS, mice were orally administered 3‐HAA in a DHEA‐induced PCOS mouse model (**Figure** **4**A). We first observed that DHEA‐treated mice exhibited reduced serum 3‐HAA levels, while these changes were reversed following 3‐HAA treatment (Figure S4A, Supporting Information). Moreover, DHEA treatment induced a distinct PCOS phenotype compared with control mice, as evidenced by insulin resistance and disrupted estrous cycles (Figure 4B–E). However, 3‐HAA administration significantly reduced plasma glucose levels in GTT and ITT assays compared with DHEA‐treated mice (Figure 4B,C), thereby improving insulin resistance. Additionally, DHEA‐treated mice receiving 3‐HAA exhibited restored estrous cycles compared with those treated with DHEA alone (Figure 4D,E). Ovaries from mice treated with DHEA displayed increased numbers of cyst‐like follicles and fewer corpora lutea relative to control mice (Figure 4F,G); these changes were reversed upon 3‐HAA administration (Figure 4F,G). Furthermore, mice in the DHEA group exhibited elevated hormone levels, including testosterone and luteinizing hormone, compared with control mice; however, these increases were attenuated in DHEA‐treated mice subjected to 3‐HAA treatment (Figure 4H). Additionally, we further confirmed that 3‐HAA treatment alleviated PCOS induced by P‐FMT in mice, as evidenced by improved ovarian morphology, estrous cycle, insulin sensitivity, and hormone levels (Figure 4I–K; Figure S4B–F, Supporting Information). Collectively, these findings indicate that 3‐HAA alleviates PCOS in mice.

### 2.5 3‐HAA Ameliorates DHEA‐Induced PCOS by Promoting De Novo Synthesis of NAD

**Figure 5:**
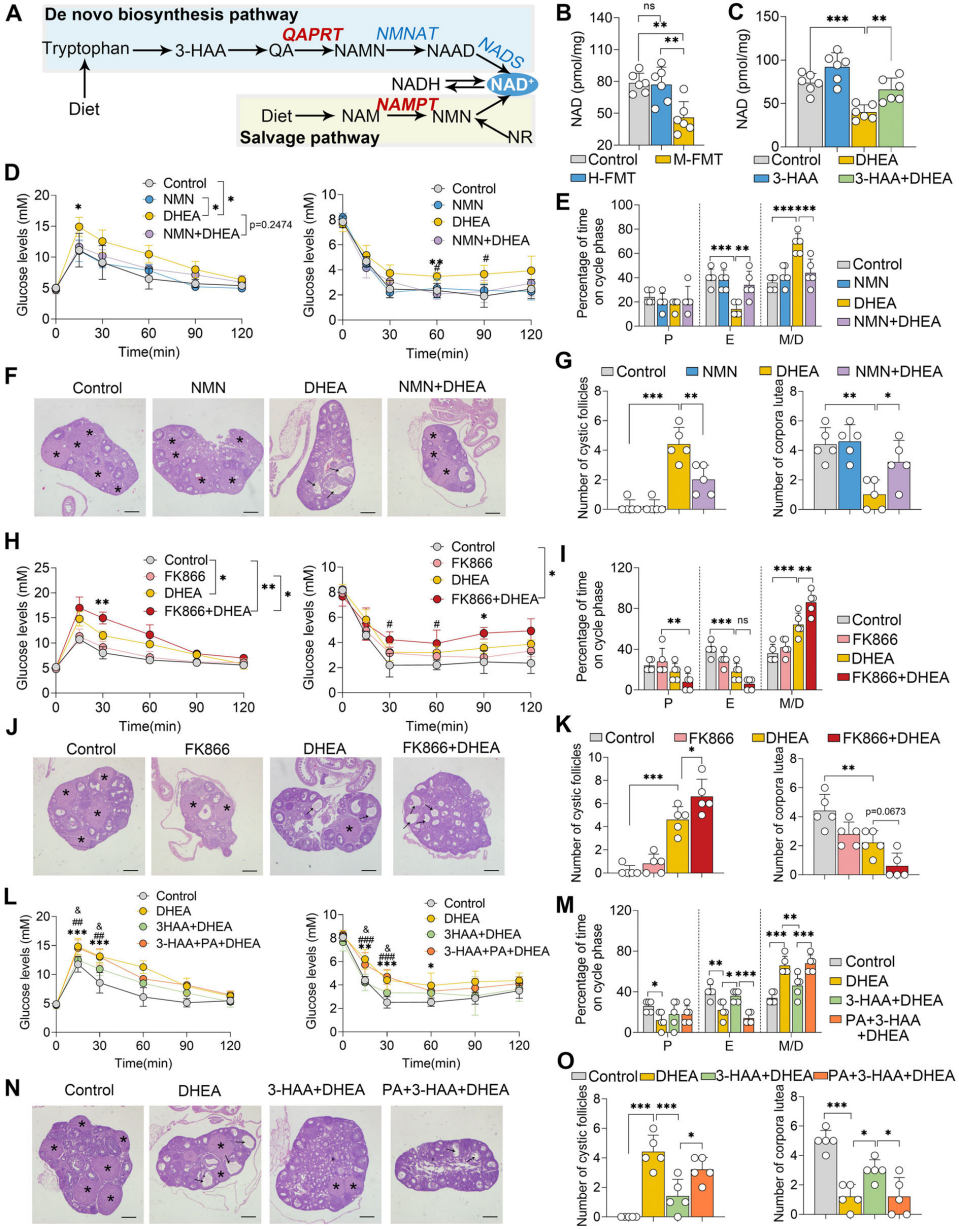
3‐HAA alleviates DHEA‐induced PCOS by enhancing the de novo synthesis of NAD. A) Schematic representation of the de novo synthesis pathway and salvage pathway for NAD synthesis. In the de novo synthesis pathway, 3‐HAA is metabolized into QA, which is subsequently converted into NAMN via *QAPRT*. NAMN is then further converted into NAAD and ultimately NAD through the actions of *NMNAT* and *NADS* enzymes. In the salvage pathway, NAM is converted into NMN by *NAMPT*, which is subsequently transformed into NAD. Notably, *QAPRT* and *NAMPT* serve as the rate‐limiting enzymes in these pathways. B,C) Analysis of ovarian NAD levels in FMT‐treated and 3‐HAA‐treated mice (n = 6). For panels D‐G, prepubertal female mice (21‐day‐old) were intraperitoneally administered NMN (500 mg kg^−1^) and subcutaneously injected with DHEA (60 mg kg^−1^) daily for 21 days. D) GTT and ITT assays were performed (n = 5). ^*^ *p* < 0.05 and ^**^ *p* < 0.01 compared to the control group. ^#^ *p* < 0.05 compared to the DHEA‐treated group. E) Determination of estrous cycles based on vaginal smears. F) Representative H&E‐stained images of ovarian tissues from indicated groups (scale bar, 200 µm). G) Quantitative analysis of cystic follicles and corpora lutea based on H&E‐stained sections (n = 5). For H–K, prepubertal (21‐day‐old) female mice were intraperitoneally administered FK866 (30 mg kg^−1^) and subcutaneously injected with DHEA (60 mg kg^−1^) daily for 21 days. H) GTT and ITT assays were performed (n = 5). ^*^ *p* < 0.05 and ^**^ *p* < 0.01 compared to the control group. ^#^ *p* < 0.05 compared to the DHEA‐treated group. I) The Estrous cycle was determined based on vaginal smears. J) Representative H&E‐stained images of ovarian tissues from the indicated groups are shown (scale bar, 200 µm). K) Quantitative analysis of cystic follicles and corpora lutea was conducted based on H&E‐stained sections (n = 5). For L–O, prepubertal (21‐day‐old) female mice were intraperitoneally treated with 3‐HAA (200 mg kg^−1^) and 200 µL of 100 µM phthalic acid (PA), and subcutaneously injected with DHEA (60 mg kg^−1^) daily for 21 days. L) GTT and ITT assays were performed (n = 5). ^*^ *p* < 0.05, ^**^ *p* < 0.01, and ^***^ *p* < 0.001 compared to the control group. ^##^ *p* < 0.01 and ^###^ *p* < 0.001 compared to the DHEA‐treated group. ^&^ *p* < 0.05 compared to the 3‐HAA+DHEA‐treated group. M) Estrous cycle was determined based on vaginal smears. N) Representative H&E‐stained images of ovarian tissues from the indicated groups are shown (scale bar, 200 µm). O) Quantitative analysis of cystic follicles and corpora lutea was conducted based on H&E‐stained sections (n = 5). Data are expressed as mean ± SD. Statistical significance was analyzed using one‐way ANOVA followed by Tukey's test for panels B, C, E, G, I, K, M, and O, and two‐way ANOVA for panels D, H, and L. ^*^ *p* < 0.05, ^**^ *p* < 0.01, and ^***^ *p* < 0.001 indicate significance.

Considering 3‐HAA as a substrate for the production of NAD^+^ (**Figure** **5**A), an indispensable redox coenzyme for host health, and given that its decline is associated with metabolic dysfunction, including PCOS,^[^ ^18^, ^34^ ^]^ we investigated whether alterations in NAD^+^ levels were involved in the protective effects of 3‐HAA against PCOS in mice. We first observed that mice subjected to P‐FMT exhibited reduced ovarian NAD^+^ levels compared to those in the Control and H‐FMT groups (Figure 5B). Patients with PCOS also exhibited reduced NAD^+^ levels in the blood (Figure S5A, Supporting Information). Additionally, DHEA‐treated mice exhibited decreased NAD^+^ levels in both the ovary and blood compared to the Control group. Conversely, 3‐HAA‐treated mice showed increased NAD^+^ levels in the ovary and blood relative to the Control group, and 3‐HAA treatment effectively reversed the DHEA‐induced reduction in NAD^+^ levels (Figure 5C; Figure S5B, Supporting Information).

To investigate the role of NAD^+^ in the development of PCOS, mice were administered nicotinamide mononucleotide (NMN), a precursor of NAD^+[^ ^34^ ^]^ (Figure 5A). NMN treatment effectively reversed the DHEA‐induced decline in ovarian NAD+ levels (Figure S5C, Supporting Information) and alleviated DHEA‐induced insulin resistance, as evidenced by reduced GTT and ITT values compared with the DHEA group (Figure 5D; Figure S5D, Supporting Information). Consistently, DHEA‐treated mice that received NMN intervention exhibited an improved estrous cycle (Figure 5E; Figure S5E, Supporting Information). Histological analysis revealed that NMN treatment mitigated the DHEA‐induced increase in cyst‐like follicles and promoted the formation of corpora lutea in the ovary (Figure 5F,G). These beneficial effects were further confirmed by the normalization of hormone profiles disrupted by DHEA, including testosterone and luteinizing hormone levels (Figure S5F, Supporting Information).

We further confirmed the protective role of NAD^+^ production in PCOS by treating mice with FK866, an inhibitor of nicotinamide phosphoribosyltransferase (*NAMPT*), which is the rate‐limiting enzyme in the NAD^+^ salvage pathway^[^ ^12^ ^]^ (Figure 5A). The results demonstrated that FK866 treatment exacerbated the DHEA‐induced reduction in NAD^+^ levels in both the ovary and blood (Figure S5G,H, Supporting Information). DHEA‐treated mice subjected to FK866 exhibited more severe insulin resistance, as evidenced by GTTs and ITTs (Figure 5H; Figure S5I, Supporting Information). Elevated levels of testosterone and luteinizing hormone were detected in DHEA‐treated mice subjected to FK866 treatment, compared with those in the DHEA‐only group (Figure S5J, Supporting Information). Additionally, impaired estrous cycles were observed in DHEA‐treated mice receiving FK866 intervention compared to those in the DHEA‐only group (Figure 5I; Figure S5K, Supporting Information). Similarly, mice treated with both FK866 and DHEA displayed a greater number of cyst‐like follicles and fewer corpora lutea in the ovaries compared to those treated with DHEA alone (Figure 5J,K).

We further examined the extent to which NAD^+^ production contributes to the protective effects of 3‐HAA on PCOS by treating 3‐HAA‐administered mice with phthalic acid (PA), an inhibitor of quinolinate phosphoribosyltransferase (*QPRT*), the rate‐limiting enzyme in de novo NAD^+^ biosynthesis^[^ ^35^ ^]^ (Figure 5A). The results showed that the protective effects of 3‐HAA were weakened by PA treatment (Figure 5L–O; Figure S5L, Supporting Information), as evidenced by increased insulin resistance (Figure 5L) and disrupted estrous cycle (Figure 5M; Figure S5L, Supporting Information), as well as changed ovarian histological changes (Figure 5N,O). Collectively, these findings suggest that the protective effects of 3‐HAA against PCOS are critically dependent on NAD^+^ production.

### 2.6 NAD^+^ Suppresses DHEA‐Induced Ferroptosis in PCOS Mice

**Figure 6:**
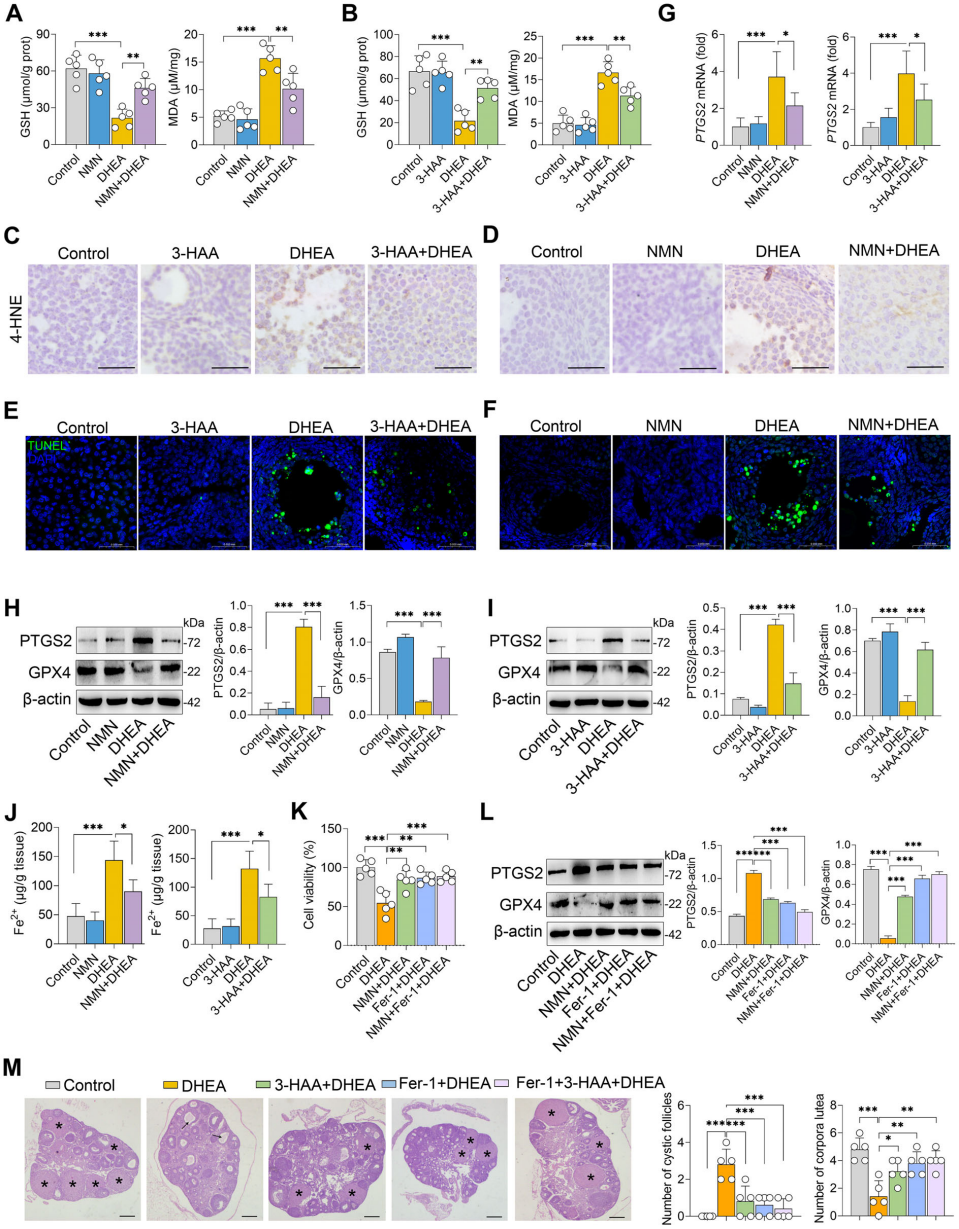
NAD^+^ inhibits DHEA‐induced ferroptosis in PCOS mice. A,B) Ovarian GSH and MDA levels in NMN‐ and 3‐HAA‐treated mice (n = 5). C,D) Representative 4‐HNE‐stained images of ovarian tissues from the indicated groups of mice (scale bar, 20 µm). E,F) Representative TUNEL‐stained images of ovarian tissues from the indicated groups of mice (scale bar, 50 µm). G) Ovarian PTGS2 mRNA expression in the indicated groups of mice (n = 5). H,I) Ovarian PTGS2 and GPX4 protein levels analyzed by western blotting in DHEA‐treated mice subjected to NMN and 3‐HAA treatments (n = 3). J) Ovarian Fe^2+^ levels in DHEA‐induced PCOS mice treated with NMN and 3‐HAA (n = 5). For K,L), KGN cells were pretreated with Fer‐1 (2 µM) and NMN (1 mM) for 2 h, followed by treatment with DHEA (20 µM) for 24 h. (K) Cell viability assay (n = 5). (L) PTGS2 and GPX4 protein levels in NMN‐ and Fer‐1‐treated KGN cells were analyzed by western blotting (n = 3). M. Prepubertal female mice (21‐day‐old) were intraperitoneally injected with 3‐HAA (200 mg kg^−1^) and Fer‐1 (10 mg kg^−1^), and subcutaneously injected with DHEA (60 mg kg^−1^) daily for 21 days. Representative H&E‐stained images of ovarian tissues from the indicated groups of mice (scale bar, 200 µm) and analysis of the number of cystic follicles and corpora lutea based on H&E‐stained sections (n = 5). Data are expressed as mean ± SD. Statistical significance was determined using one‐way ANOVA followed by Tukey's test (A, B, and G‐M). ^*^ *p* < 0.05, ^**^ *p* < 0.01, and ^***^ *p* < 0.001 indicate significance.

As NAD^+^ is an essential coenzyme in redox reactions and has been reported to regulate lipid peroxidation and ferroptosis,^[^ ^12^ ^]^ we next investigated whether the protective effects of 3‐HAA and NAD^+^ are associated with the inhibition of ferroptosis in PCOS. We first demonstrated that DHEA treatment decreased ovarian glutathione (GSH) levels compared to the control group, while this reduction was reversed by NMN and 3‐HAA treatments (**Figure** **6**A,B). Furthermore, the increased ovarian MDA levels induced by DHEA were also reversed by NMN and 3‐HAA treatments (Figure 6A,B). Consistently, DHEA significantly increased ovarian 4‐hydroxynonenal (4‐HNE) levels, which were reversed by 3‐HAA and NMN treatments (Figure 6C,D). Moreover, mice treated with DHEA exhibited increased ovarian cell death compared to control mice, whereas 3‐HAA and NMN attenuated DHEA‐induced cell death (Figure 6E,F). Mice treated with DHEA exhibited elevated ovarian PTGS2 mRNA expression compared to control mice, which was attenuated by NMN and 3‐HAA treatments (Figure 6G). Additionally, NMN and 3‐HAA treatments restored the changes in PTGS2 and GPX4 expression caused by DHEA (Figure 6H,I). Moreover, NMN and 3‐HAA treatments reduced the accumulation of Fe^2+^ induced by DHEA (Figure 6J), suggesting that NMN and 3‐HAA inhibit ferroptosis in PCOS mice.

In vitro, DHEA treatment significantly decreased cell viability, and this reduction was partially rescued by NMN and ferrostatin‐1 (Fer‐1), a specific ferroptosis inhibitor (Figure 6K). However, no additional effects were observed in cells co‐treated with NMN and Fer‐1 compared to those treated with NMN or Fer‐1 alone (Figure 6K). Moreover, Fer‐1 treatment mitigated the DHEA‐induced increase in PTGS2 expression and restored GPX4 levels in KGN cells. Nevertheless, no further alterations in PTGS2 or GPX4 expression were detected in Fer‐1‐treated cells upon NMN administration (Figure 6L). In vivo, DHEA‐treated mice were subjected to 3‐HAA and Fer‐1 treatments. We found that Fer‐1 treatment improved insulin resistance (Figure S6A,B, Supporting Information), normalized the estrous cycle (Figure S6C,D, Supporting Information), and alleviated ovarian damage (Figure 6M) in DHEA‐treated mice. However, no enhanced protective effects were observed in mice co‐treated with both Fer‐1 and 3‐HAA (Figure 6M; Figure S6A–D, Supporting Information). Collectively, these findings indicate that the protective effects of 3‐HAA and NAD^+^ against PCOS are closely associated with the inhibition of ferroptosis.

### 2.7 NAD Suppresses Ferroptosis by Inhibiting the cGAS‐STING Pathways

**Figure 7:**
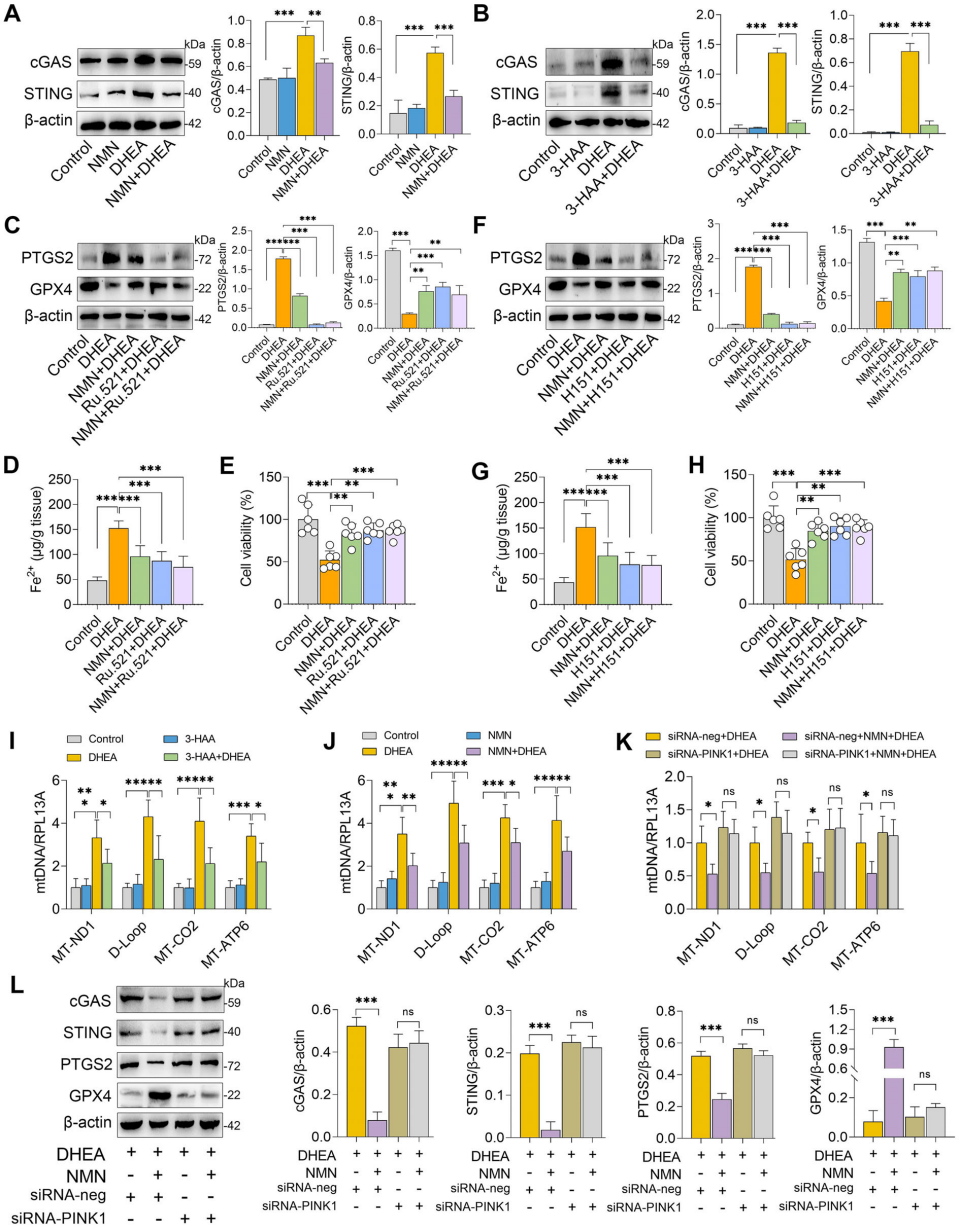
NAD suppresses ferroptosis through inhibiting cGAS‐STING pathways. A,B) Ovarian cGAS and STING protein expressions analyzed by western blotting in DHEA‐treated mice subjected to NMN and 3‐HAA treatments (n = 3). For panels C–E, cells were pretreated with Ru.521 (100 µM) and NMN (1 mM) for 2 h, followed by treatment with DHEA (20 µM) for an additional 24 h. C) Western blot analysis of PTGS2 and GPX4 protein expressions (n = 3). D) Intracellular Fe^2+^ levels measured in different treatment groups (n = 5). E) Cell viability assessed using a cell viability assay (n = 5). For panels F‐H, cells were pretreated with H151 (2 µM) and NMN (1 mM) for 2 h, followed by treatment with DHEA (20 µM) for an additional 24 h. F) Western blot analysis of PTGS2 and GPX4 protein expressions in NMN‐ and H151‐treated cells (n = 3). G) Intracellular Fe^2+^ levels in H151‐ and NMN‐treated cells (n = 5). H) Cell viability assessed using a cell viability assay (n = 5). I,J) DNA was extracted from cytoplasmic and nuclear fractions of 3‐HAA‐ and NMN‐treated cells under DHEA treatment conditions, and mtDNA/RPL13A levels were analyzed by qPCR (n = 5). K and L. Cells were transfected with siRNA‐PINK1 for 48 h, followed by treatment with NMN and DHEA. K) DNA was extracted from cytoplasmic and nuclear fractions, and mtDNA/RPL13A levels were analyzed by qPCR. L) Western blot analysis of cGAS, STING, PTGS2, and GPX4 protein expression levels (n = 3). Data are presented as mean ± SD. ^*^ *p* < 0.05, ^**^ *p* < 0.01, and ^***^ *p* < 0.001, determined by one‐way ANOVA followed by Tukey's post hoc test (A–L).

Depletion of NAD^+^ was closely associated with mitochondrial homeostasis and cellular senescence in multiple organs, including the ovary.^[^ ^17^, ^19^ ^]^ This process was regulated through the cyclic GMP‐AMP synthase (cGAS) and stimulator of interferon genes (STING) pathway by recognizing intracellular DNA.^[^ ^28^ ^]^ Additionally, activation of the cGAS‐STING pathway was linked to elevated ferroptosis.^[^ ^23^ ^]^ Therefore, we next investigated whether NAD^+^ inhibited ferroptosis through regulation of the cGAS‐STING pathway. We first observed that increased protein expression in the cGAS‐STING pathway was detected in DHEA‐treated mice, but these effects were reversed by NMN and 3‐HAA treatments (**Figure** **7**A,B). To confirm the role of the cGAS‐STING pathway in NAD^+^‐mediated inhibition of ferroptosis, specific inhibitors of cGAS (Ru.521) and STING (H151) were employed. The results demonstrated that administration of Ru.521 and NMN reduced PTGS2 expression and increased GPX4 levels in DHEA‐treated cells. However, no significant differences were observed in Ru.521 and NMN co‐treated cells compared with those treated with Ru.521 and NMN alone (Figure 7C). Consistently, intracellular Fe^2+^ concentrations were reduced in DHEA‐treated cells subjected to Ru.521 and NMN treatments, while Fe^2+^ levels remained stable in Ru.521 and NMN co‐treated cells compared with those treated with Ru.521 and NMN alone (Figure 7D). Furthermore, we found that although both NMN and Ru.521 improved cell viability impaired by DHEA, no additional effects were observed in Ru.521 and NMN co‐treated cells (Figure 7E). Similarly, inhibition of STING by H151 also yielded consistent results (Figure 7F–H). Impaired NAD^+^ production can increase the release of mitochondrial DNA (mtDNA) by disrupting mitophagy, leading to enhanced activation of the cGAS‐STING pathway.^[^ ^17^, ^28^ ^]^ We then investigated whether mtDNA release is involved in NAD+‐mediated inhibition of cGAS‐STING activation and ferroptosis. As expected, DHEA treatment increased mtDNA levels in cells, as evidenced by elevated expression of mitochondrial genes (*MT‐ND1*, *D‐Loop*, *MT‐CO2*, and *MT‐ATP6*) relative to the nuclear gene *RPL13A*,^[^ ^17^, ^28^ ^]^ while these increases were reversed by 3‐HAA and NMN treatments (Figure 7I,J). Consistently, we found that DHEA treatment impaired mitochondrial morphology, as indicated by increased mitochondrial matrix electron density, along with compromised mitochondrial membranes and cristae, effects that were reversed by NMN treatment (Figure S6E, Supporting Information). We next knocked down the PTEN‐induced kinase 1 (PINK1) gene (Figure S6F, Supporting Information), an important regulator of mitophagy and mtDNA release,^[^ ^17^, ^28^ ^]^ and found that siRNA‐PINK1 treatment weakened NMN's inhibitory effects on mtDNA release (Figure 7K), and compromised NMN's protective effects against cGAS‐STING activation and ferroptosis (Figure 7L). Collectively, these findings suggest that NMN inhibits DHEA‐induced ferroptosis via regulation of the cGAS‐STING pathways.

## 3 Discussion

PCOS is a serious condition that affects female ovarian health and increases the risk of various diseases, including endometrial cancer. The pathogenesis of PCOS remains poorly understood, which has hindered the development of effective preventive and therapeutic strategies. Recent studies have highlighted the involvement of gut microbiota and microbial metabolism in multiple diseases. In this study, we observed distinct gut microbial compositions in PCOS patients, which were closely associated with PCOS development. Specifically, P‐FMT from PCOS patients to mice induced PCOS‐related symptoms and histological changes. Importantly, both PCOS patients and P‐FMT recipient mice exhibited significantly altered metabolic profiles in the gut, with many shared differential metabolites between donors and recipients. These findings suggest a gut microbiota‐mediated metabolic alteration in PCOS. Furthermore, we identified impaired tryptophan metabolism, particularly reduced levels of 3‐HAA, in both PCOS patients and P‐FMT mice. Administration of 3‐HAA to mice alleviated DHEA‐induced PCOS symptoms. Mechanistically, 3‐HAA promoted NAD production via the de novo biosynthesis pathway, thereby inhibiting DHEA‐induced ferroptosis through regulation of the cGAS‐STING axis.

The gut microbiota has been associated with PCOS. However, variations in the gut microbiota of PCOS patients exhibited significant differences across studies. Consistent with previous studies,^[^ ^2^, ^6^, ^9^ ^]^ we observed reduced alpha diversity of the gut microbiota in PCOS patients. Furthermore, our findings revealed increased relative abundances of *Proteobacteria* and *Enterobacter* in PCOS patients, while commensal probiotics such as *Lactobacillus*, *Prevotella*, *Roseburia*, and *Alistipes* were significantly depleted. These results align with prior observations that PCOS individuals exhibit increased *Enterobacteriaceae* and reduced short‐chain fatty acid (SCFA) producers, including *Prevotella*.^[^ ^36^ ^]^ Additionally, decreased *Lactobacillus* abundances have been reported in PCOS models.^[^ ^37^, ^38^ ^]^ Treatment with *L. reuteri* demonstrated protective effects against PCOS in rats.^[^ ^7^ ^]^ Similarly, the role of the gut microbiota in PCOS pathogenesis was confirmed through FMT, where Lactobacillus and *Alistipes* abundances were depleted in P‐FMT mice. Other taxa including Other taxa, including *Parasutterella* and *Muribaculaceae*, were also depleted in P‐FMT mice compared with those in the H‐FMT group. *Muribaculaceae* has been associated with serum testosterone levels and glucose metabolism in PCOS.^[^ ^39^ ^]^ Additionally, *Parasutterella* has been linked to lipid metabolism, inflammatory responses, and tryptophan metabolism.^[^ ^40^, ^41^ ^]^ However, the roles of these microorganisms in the pathogenesis of PCOS remain to be elucidated. Qi et al. identified significantly increased fecal Bacteroides abundances in PCOS patients and demonstrated the role of *Bacteroides vulgatus* in promoting PCOS development via bile acid metabolism regulation.^[^ ^2^ ^]^ Meanwhile, María et al., reported that *Catenibacterium* and *Kandleria* were enriched in the gut microbiota of PCOS patients.^[^ ^9^ ^]^ Nevertheless, these findings were not replicated in our study, highlighting the heterogeneity of the gut microbiota in PCOS patients and the complex interplay of multiple factors influencing this condition.

Metabolic changes represent one of the most common and direct mechanisms underlying the interaction between gut microbiota and the host. Our findings revealed that patients with PCOS exhibited distinct metabolic profiles and numerous differential metabolites, some of which were also observed in recipient mice, suggesting a gut microbiota‐mediated metabolic alteration. Consistent with previous studies,^[^ ^2^ ^]^ bile acid metabolism was altered in PCOS patients; however, this change was not transferred to FMT recipient mice. Among the shared differential metabolites between donor and recipient mice, we identified multiple metabolites associated with tryptophan metabolism, including 3‐HAA, 5‐HT, and FK. Treatment with 3‐HAA alleviated DHEA‐induced PCOS in mice. A prior study demonstrated that 3‐HAA could attenuate atherosclerosis and improve plasma lipid metabolism, indicating its potential protective role in metabolic diseases.^[^ ^42^ ^]^ Additionally, another study showed that 3‐HAA metabolism regulates hepatic sterol regulatory element‐binding protein‐2 (SREBP‐2) activity and inhibits inflammasome activation in macrophages.^[^ ^43^ ^]^

Apart from its direct anti‐inflammatory effects, 3‐HAA serves as a substrate for de novo NAD biosynthesis.^[^ ^11^, ^12^ ^]^ Previous studies have demonstrated that disrupted NAD metabolism is associated with PCOS.^[^ ^18^, ^19^ ^]^ Indeed, we observed that both P‐FMT and DHEA‐treated mice exhibited reduced NAD levels; however, administration of 3‐HAA restored the DHEA‐induced decrease in NAD. Consistent with prior findings,^[^ ^18^ ^]^ treatment of mice with the NAD precursor NMN alleviated DHEA‐induced PCOS symptoms, whereas inhibition of NAD production via NAMPT blockade exacerbated these symptoms. In line with our results, previous studies also revealed that NAD improves insulin resistance and ameliorates diabetes and fatty liver disease,^[^ ^15^, ^44^ ^]^ as well as regulates inflammation.^[^ ^44^ ^]^ Furthermore, impaired NAD production accelerates ovarian aging.^[^ ^19^, ^45^ ^]^

We further discovered that 3‐HAA and NAD restrict DHEA‐induced ferroptosis. Ferroptosis is a newly identified form of regulated cell death characterized by lipid peroxidation and Fe^2+^ accumulation, which has recently been implicated in the pathogenesis of PCOS.^[^ ^4^ ^]^ The induction of ferroptosis by RSL3 resulted in an increased number of cyst‐like follicles, whereas the inhibition of ferroptosis using Fer‐1 improved the PCOS profiles induced by DHEA.^[^ ^4^ ^]^ Additionally, NAD^+^ has been shown to modulate ferroptosis through the regulation of the NADH‐FSP1‐CoQ10 pathway.^[^ ^46^ ^]^ Furthermore, the disruption of NAD^+^ biosynthesis exacerbated lipid peroxidation and tissue damage.^[^ ^12^ ^]^ Supplementation with NMN to boost NAD levels effectively attenuated ferroptosis in multiple organs, including the ovaries.^[^ ^47^, ^48^ ^]^

Iron‐dependent lipid peroxidation is not solely caused by the reduction of cellular antioxidant capacity but is also induced by ferritin degradation.^[^ ^24^ ^]^ The cGAS‐STING pathway serves as a critical regulator of autophagy and ferroptosis.^[^ ^36^ ^]^ We further observed that PCOS mice exhibited elevated expression levels of the cGAS‐STING pathway, which were suppressed by NMN and 3‐HAA. Consistent with prior findings, studies have demonstrated that NAD production restricts the activation of the cGAS‐STING pathway.^[^ ^17^, ^28^ ^]^ Pharmacological inhibition of the cGAS‐STING pathway improved DHEA‐induced PCOS, supporting its involvement in insulin resistance and metabolic disorders.^[^ ^49^, ^50^ ^]^ Increased STING activation has been reported to facilitate FTH degradation via NCOA4‐mediated ferritinophagy.^[^ ^36^, ^51^ ^]^ Patients with PCOS also exhibited reduced FTH levels, and DHT enhanced NOCA4‐dependent ferritinophagy during PCOS.^[^ ^4^ ^]^ Moreover, STING can directly bind to ACSL4 and promote lipid peroxidation,^[^ ^52^ ^]^ which was observed in DHEA‐induced PCOS in mice.^[^ ^53^ ^]^ Additionally, we found that both 3‐HAA and NMN inhibited the release of mtDNA, consistent with previous studies showing that disrupted NAD^+^ production impaired mitophagy and promoted cGAS‐STING activation by elevating mtDNA levels.^[^ ^17^, ^28^ ^]^ Disrupted mitochondrial functions were also detected in mice with PCOS.^[^ ^54^ ^]^ Interestingly, studies have demonstrated that NAD^+^ can influence the functions of multiple organs,^[^ ^55^, ^56^ ^]^ and interactions between organs, such as the liver and kidneys, can collaboratively regulate NAD^+^ levels.^[^ ^55^ ^]^ Specifically, Feng et al. demonstrated that restoring NAD^+^ levels alleviated busulfan‐induced ferroptosis by modulating the SIRT2‐PGC‐1α/ACLY pathways.^[^ ^26^ ^]^ Pretreatment with NMN mitigated cadmium‐induced renal cell ferroptosis by reducing mitochondrial GPX4 acetylation.^[^ ^27^ ^]^ Feng et al. also showed that NMN recruits GSH to inhibit ferroptosis, thereby alleviating skin injury,^[^ ^57^ ^]^ suggesting that multiple mechanisms may be involved in the process of NAD^+^ against ferroptosis. Notably, while our study highlights the beneficial role of 3‐HAA‐mediated de novo NAD^+^ biosynthesis in preventing PCOS, we cannot rule out the possibility that 3‐HAA alleviates PCOS through other pathways, and multiple pathways may synergistically contribute to its effects. Additionally, we acknowledge that other gut microbiota‐associated metabolites may also play a role in the pathogenesis of PCOS.

Collectively, our results demonstrate that PCOS patients exhibit dysbiosis in gut microbiota and metabolism, particularly characterized by reduced levels of 3‐HAA, which contributes to the impairment of NAD^+^ biosynthesis. Administration of 3‐HAA or NMN alleviates PCOS symptoms by inhibiting ferroptosis, with the underlying mechanism being associated with the suppression of the cGAS‐STING axis. Our study not only elucidates new mechanisms underlying PCOS pathogenesis mediated by gut microbiota but also highlights that modulating gut microbiota and enhancing NAD synthesis may represent promising strategies for the prevention and treatment of PCOS.

## 4 Experimental Section

### Human Subjects

The study was approved by the China‐Japan Union Hospital of Jilin University (2024032105). The 2003 Rotterdam criteria were used to distinguish between PCOS patients and healthy subjects. It required at least 2 of the following 3 symptoms to be diagnosed as PCOS: a) Oligo‐ and/or anovulation; b) Clinical and/or biochemical signs of hyperandrogenism; c) polycystic ovaries. In addition, it needed exclusion of other aetiologies (congenital adrenal hyperplasias, androgen‐secreting tumors, Cushing's syndrome) and no antibiotics, probiotics, or hormone drugs within a month, no pregnancy or breastfeeding within a year. A total of 39 patients with PCOS and 36 healthy individuals were recruited. All participants in this study obtained informed written consent from themselves or their close relatives. All individuals with PCOS were first‐visit patients and had not received PCOS‐related treatment. Height, body weight, hormone levels, and biochemical markers were determined as previously.^[^ ^2^ ^]^

### Animals and Treatments

The animal experiments were approved by the Institutional Animal Care and Use Committee (IACUC) of Jilin University (SY202501020). Female C57BL/6 mice at prepuberal (21‐day‐old) were obtained from Liaoning Changsheng biotechnology co., Ltd. (Benxi, China) and maintained at a 12 h light and dark cycle with free drinking water and diet. For the FMT experiment, five donors were randomly selected from PCOS patients and healthy controls, and fecal samples were treated as previously.^[^ ^48^ ^]^ Briefly, fecal samples from donors of the same groups were weighed and mixed, followed by sterile PBS dissolution (100 mg mL^−1^). After centrifuging at 100 × g for 5 min, fecal supernatants were collected for FMT. The preparation of FMT samples was carried out in sterile anaerobic incubators to reduce the loss of microbes. Mice were treated with an antibiotic cocktail (ABX, 200 mg kg^−1^ ampicillin, neomycin, and metronidazole, and 100 mg kg^−1^ vancomycin) for five consecutive days to deplete commensal microbes.^[^ ^29^, ^48^ ^]^ After replacing ABX with water for one day and then followed by FMT. ABX‐treated mice were randomly divided into three groups: 1) P‐FMT group: mice were orally treated with 100 µL fecal supernatants for PCOS patients; 2) H‐FMT group: mice were orally treated with 100 µL fecal supernatants for healthy controls; 3) Control group: mice were treated with 100 µL PBS. All mice underwent FMT for three consecutive days after replacing ABX, and then performed once every two days for a total of 21 days.

For the DHEA‐induced PCOS model, female mice at prepuberal (21‐day‐old) were subcutaneously injected with DHEA (60 mg kg^−1^ body weight) daily for 21 days.^[^ ^2^, ^58^, ^59^ ^]^ Mice from the control group were treated with 200 µL of sesame oil. For 3‐HAA treatment, mice were treated with 200 mg kg^−1^ 3‐HAA intraperitoneally daily for 21 days.^[^ ^42^, ^60^ ^]^ For NMN treatment, mice were intraperitoneally treated with 500 mg kg^−1^ daily for 21 days.^[^ ^61^, ^62^ ^]^ For FK866 treatment, mice were treated with 30 mg kg^−1^ FK866 daily for 21 days intraperitoneally.^[^ ^12^, ^16^ ^]^ For the QAPRT inhibition experiment, mice were treated with 200 µL of 100 µM phthalic acid intraperitoneally with 3‐HAA treatment daily for 21 days.^[^ ^63^ ^]^ For the Fer‐1 treatment experiment, mice were treated with 10 mg kg^−1^ Fer‐1 with or without 3‐HAA daily for 21 days.^[^ ^64^ ^]^ For all treatments, drugs were administered for 2 h before DHEA. After 21 days, the ovarian, liver, and serum were collected for analysis.

### Cell Culture and Treatments

KGN cells were purchased from American Type Culture Collection (ATCC) and cultured in F12 medium supplemented with 10% fetal bovine serum and 1% antibiotics (penicillin and streptomycin) at 37 °C with 5% CO_2_. For all treatments, cells were incubated in six‐well plates overnight and then incubated with an antibiotic‐free medium. For NMN treatment, prepared cells were treated with NMN (1 mM) for 2 h,^[^ ^61^ ^]^ followed by DHEA (20 µM) treatment for the next 24 h.^[^ ^65^ ^]^ For ferroptosis, cGAS and STING inhibition in vitro, cells were treated with Fer‐1 (2 µM),^[^ ^64^ ^]^ Ru.521 (100 µM),^[^ ^66^ ^]^ and H151 (2 µM)^[^ ^66^ ^]^ with or without NMN (1 mM) for 2 h, respectively, followed by DHEA (20 µM) treatment for the next 24 h. For the siRNA transfection experiment, PINK1‐specific siRNA and negative control siRNA (Sangon Biotech, China) were transfected into cells using Lipo8000 Transfection Reagent according to the manufacturer's protocol. The siRNA was diluted in Opti‐MEM Medium to a final concentration of 25 nM. NMN (1 mM) was added 48 h after siRNA transfection. Subsequently, cells were treated with DHEA (20 µM) for 24 h, and then harvested for analysis.

### Vaginal Smears and Estrous Cycle Determination

Vaginal smears and estrous cycle determination were performed as previously.^[^ ^2^ ^]^ In brief, vaginal sears were performed daily from the 10th to the 19th day after the first day of FMT or DHEA treatment. According to the predominant cell types in vaginal smears, the stage of the estrous cycle was identified by microscopic analysis based on Shorr staining. The stage of the estrous cycle includes proestrus (P, round, nucleated epithelial cells), estrus (E, cornified squamous epithelial cells), metestrus (M, epithelial cells and leukocytes), and diestrus (D, nucleated epithelial cells and a predominance of leukocytes).

### GTT and ITT

For the GTT experiment, mice at the 21st day after the first day of FMT or DHEA treatment were fasted for 12 h. For ITT, mice were fasted for 4 h. Mice were determined fasting glucose levels and then injected intraperitoneally with 2 mg kg^−1^ D‐glucose for GTT, or 1 IU kg^−1^ insulin for ITT. Next, tail vein blood sampling was performed at 15, 30, 60, 90, and 120 min after D‐glucose or insulin injection, and glucose levels were determined by a blood glucose Accu‐Chek Performa (Roche Diagnostics).^[^ ^2^ ^]^

### Serum Biochemical Assays

The blood samples were collected on the 21st day and centrifuged at 3000 × g for 15 min at 4 °C. Serum testosterone (DEV9911; Demeditec Diagnostics), estradiol (108667; Abcam), and luteinizing hormone (H206‐96T; NanJing Jian Cheng Bioengineering Institute) levels were detected according to the manufacturer's instructions.

### Histological Analysis

Tissues used for histological analysis were collected and fixed with 4% paraformaldehyde. After dehydration and paraffin embedding, 5 µm slices were prepared and stained with hematoxylin and eosin (H&E) staining. Histological changes were determined by using an optical microscope (Olympus, Tokyo, Japan).

### Fertility Assessment

Female mice were mixed with male mice at a ratio of 1:1 at the end of FMT, and the number of the first litter of pups was quantified.

### Iron Assay

Intracellular Fe^2+^ levels were detected using a commercialized iron assay kit according to the manufacturer's instructions.

### GSH, MDA, and NAD Assays

GSH, MDA, and NAD levels were detected by GSH (A006‐2, Nanjing Jiancheng Bioengineering Institute, China), MDA (MAK085, Sigma‐Aldrich, USA), and NAD assay kits according to the manufacturer's instructions.

### Immunohistochemistry

The paraffin‐embedded ovarian sections were deparaffinized, rehydrated, and subjected to antigen retrieval. Subsequently, the sections were blocked with hydrogen peroxide and 5% bovine serum albumin (BSA), incubated overnight at 4 °C with the primary antibody against 4‐hydroxynonenal (4‐HNE; MedChemExpress, HY‐P81208; diluted 1:400). On the following day, the sections were washed, incubated with the corresponding secondary antibodies, visualized with 3,3′‐diaminobenzidine (DAB) staining, counterstained with hematoxylin, and mounted. The resulting images were analyzed using an optical microscope (Olympus, Tokyo, Japan).

### Immunofluorescence

For the ovarian cell death assay, dewaxed and hydrated tissue sections were stained using a commercial terminal deoxynucleotidyl transferase dUTP nick end labeling (TUNEL) assay kit and examined under confocal fluorescence microscopy (Olympus, Tokyo, Japan).^[^ ^64^ ^]^ Apoptotic cells were labeled in green, and nuclei were stained with DAPI.

### Transmission Electron Microscopy (TEM) Assays

TEM was performed for mitochondrial observation as previously described.^[^ ^67^ ^]^ Briefly, the cells were treated as mentioned above, collected, and fixed with 2.5% glutaraldehyde, followed by post‐fixation in 1% osmium tetroxide. The samples were then dehydrated through a graded series of ethanol and acetone. Subsequently, the pellets were embedded in Epon resin (Electron Microscopy Sciences, Hatfield, PA, USA), ultrathin sections were prepared, and the sections were stained with lead citrate and uranyl acetate. Electron micrographs were captured and analyzed using a JEM‐1400 Plus transmission electron microscope (JEOL Ltd., Tokyo, Japan).

### Untargeted Metabolomics

Fecal metabolomics were performed by UHPLC‐MS/MS. In brief, 50 mg fecal samples were accurately weighed, and the metabolites were extracted using a 400 µL methanol:water (4:1, v/v) solution with 0.02 mg mL^−1^ L‐2‐chlorophenylalanin as internal standard. The mixture was allowed to settle at −10 °C and treated by High‐throughput tissue crusher Wonbio‐96c (Shanghai Wanbo Biotechnology Co., LTD) at 50 Hz for 6 min, then followed by ultrasound at 40 kHz for 30 min at 5 °C. The samples were placed at −20 °C for 30 min to precipitate proteins. After centrifugation at 13 000 × g at 4 °C for 15 min, the supernatants were carefully transferred to sample vials for LC‐MS/MS analysis. The instrument platform for LC‐MS analysis is the UHPLC‐Q Exactive system of Thermo Fisher Scientific. Chromatographic conditions:2 µL of sample was separated by an HSS T3 column (100 mm × 2.1 mm i.d., 1.8 µm) and then entered into mass spectrometry detection. The mobile phases consisted of 0.1% formic acid in water:acetonitrile (95:5, v/v) (solvent A) and 0.1% formic acid in acetonitrile:isopropanol:water (47.5:47.5:5, v/v) (solvent B). The solvent gradient changed according to the following conditions: from 0 to 0.1 min, 0% B to 5% B; from 0.1 to 2 min, 5% B to 25% B; from 2 to 9 min, 25% B to 100% B; from 9 to 13 min, 100% B to 100% B; from 13 to 13.1 min, 100% B to 0% B; from 13.1 to 16 min, 0% B to 0% B for equilibrating the systems. The sample injection volume was 2 µL, and the flow rate was set to 0.4 mL min^−1^. The column temperature was maintained at 40 °C. MS conditions: The mass spectrometric data were collected using a Thermo UHPLC‐Q Exactive Mass Spectrometer equipped with an electrospray ionization (ESI) source operating in either positive or negative ion mode. The optimal conditions were set as follows: heater temperature, 400 °C; Capillary temperature, 320 °C; sheath gas flow rate, 40 arb; Aux gas flow rate, 10 arb; ion‐spray voltage floating (ISVF), −2800 V in negative mode and 3500 V in positive mode, respectively; Normalized collision energy, 20‐40‐60 V rolling for MS/MS. Full MS resolution was 70 000, and MS/MS resolution was 17 500. Data acquisition was performed with the Data Dependent Acquisition (DDA) mode. The detection was carried out over a mass range of 70–1050 m/z.

After the mass spectrometry detection, the raw data of LC/MS are preprocessed by Progenesis QI (Waters Corporation, Milford, USA). Perform variance analysis on the matrix file after data preprocessing. The R package ropls (Version 1.6.2) performed principal component analysis (PCA) and orthogonal least partial squares discriminant analysis (OPLS‐DA), and used 7‐cycle interactive validation to evaluate the stability of the model. In addition, students *t*‐test and fold difference analysis were performed. The selection of significantly different metabolites was determined based on the Variable importance in the projection (VIP) obtained by the OPLS‐DA model and the p‐value of students *t*‐test, and the metabolites with VIP > 1, *p* < 0.05 were significantly different metabolites. Differential metabolites among the two groups were summarized and mapped into their biochemical pathways through metabolic enrichment and pathway analysis based on database search (KEGG, http://www. genome.jp/kegg/). The data were analyzed through the free online platform of the majorbio cloud platform (cloud.majorbio.com).

### Bacterial DNA Extraction and Sequencing

Microbial genomic DNA was extracted from human and mouse fecal samples using the FastDNASpin Kit for Soil (MP Biomedicals, USA) according to the manufacturer's instructions. The DNA extract was checked on 1% agarose gel, and DNA concentration and purity were determined with a NanoDrop 2000 UV–vis spectrophotometer (Thermo Fisher Scientific, MA, USA). The hypervariable region V3‐V4 of the bacterial 16S rRNA gene was amplified with primer pairs 338F (5′‐ACTCCTACGGGAGGCAGCAG‐3′) and 806R (5′‐GGACTACHVGGGTWTCTAAT‐3′) by an ABI GeneAmp 9700 PCR thermocycler (ABI, CA, USA). The PCR amplification of the 16S rRNA gene was performed as mentioned previously.^[^ ^30^ ^]^ PCR reactions were performed in triplicate, and the PCR product was extracted from 2% agarose gel and purified using the AxyPrep DNA Gel Extraction Kit (Axygen Biosciences, CA, USA) according to the manufacturer's instructions and quantified using the Quantus Fluorometer (Promega, USA). Purified amplicons were pooled in equimolar and paired‐end sequenced on an Illumina MiSeq PE300 platform/NovaSeq PE250 platform (Illumina, San Diego, USA) by Majorbio Bio‐Pharm Technology Co. Ltd. (Shanghai, China). OTUs with a 97% similarity cutoff were clustered using UPARSE version 7.1, and chimeric sequences were identified and removed. The taxonomy of each OTU representative sequence was analyzed by RDP Classifier version 2.2 against the 16S rRNA database using a confidence threshold of 0.7. Principal coordinate analysis (PCoA) was used to identify microbial structure, and linear discriminant analysis effect size (LEfSe) was performed to identify bacterial taxa that were differentially enriched in different treatment groups.

### RNA Extraction and qPCR

Total RNA was extracted using an RNA extraction kit (K0732, Thermo Scientific, USA). Briefly, cells were extracted with 1 mL Trizol and then underwent treatment with chloroform, isopropanol, and 75% ethyl alcohol under RNase‐free conditions. After RNA extraction, complementary DNA (cDNA) was synthesized using TransStart Tip Green qPCR SuperMix (TransGen Biotech, Beijing, China), Quantitative RT–PCR was performed with specific primers using a FastStart Universal SYBR Green Master Mix (ROX) (Roche, Switzerland, Basel) in a Step One Plus apparatus (Applied Biosystems, Foster City, CA, USA). Specific primer sequences used in this study were as follow: *PTGS2* (sense 5′‐TTCCAATCCATGTCAAAACCGT‐3′, antisense 5′‐AGTCCGGGTACAGTCACACTT‐3′), *MT‐ND1* (forward: 5′‐CACCCAAGAACAGGGTTTGT‐3′ and reverse: 5′‐TGGCCATGGGTATGTTGTTAA‐3′), *D‐Loop* (forward: 5′‐CTATCACCCTATTAACCACTCA‐3′ and reverse: 5′‐TTCGCCTGTAATATTGAACGTA‐3′), *MT‐CO2* (forward: 5′‐AATCGAGTAGTACTCCCGATTG‐3′ and reverse: 5′‐TTCTAGGACGATGGGCATGAAA‐3′), *MT‐ATP6* (forward: 5′‐AATCCAAGCCTACGTTTTCACA‐3′ and reverse: 5′‐AGTATGAGGAGCGTTATGGAGT‐3′), *RPL13A* (forward: 5′‐GCCCTACGACAAGAAAAAGCG‐3′ and reverse: 5′‐TACTTCCAGCCAACCTCGTGA‐3′) and *GAPDH* (forward: 5′‐AACTTTGGCATTGTGGAAGG‐3′ and reverse: 5′‐ACACATTGGGGGTAGGAACA‐3′). The reaction conditions were as follows: 52 °C for 2 min, 95 °C for 10 min, 95 °C for 15 s, and 60 °C for 1 min for 45 cycles.^[^ ^68^ ^]^ *GAPDH* and *RPL13A* served as an endogenous control. The 2^−ΔΔCt^ method was employed to calculate the relative expression of genes by calibrating with the control group.

### Western Blotting

Ovarian tissues or prepared cells were collected and used for protein extraction (Thermo Fisher Scientific, USA). The concentration of total protein was measured by the BCA Protein Assay Kit (Thermo Fisher Scientific, USA). To separate the targeted protein, SDS‐PAGE (polyacrylamide gel electrophoresis) was performed based on the protein molecular size. Targeted proteins were next bonded to 0.45 µm PVDF membranes and blocked by 5% skim milk for 3 h at room temperature. Next, specific primary antibodies including GPX4 (1:1000, DF6701, Affinity Biosciences, USA), PTGS2 (1:1000, AF7003, Affinity Biosciences, USA), cGAS (1:1000, #79978, Cell Signaling Technology, USA), STING (1:1000, DF12090, Affinity Biosciences, USA) and β‐actin (1:2000, AF7018, Affinity Biosciences, USA) were used for protein determination by incubation overnight at 4 °C. After washing with PBST for three times per 10 min to remove unbonded antibodies, the PVDF membranes were incubated with secondary antibodies (goat anti‐rabbit or rabbit anti‐mouse IgG) for 2 h at room temperature. After washing with PBST three times, the protein levels were determined using an ECL plus western blotting Detection System (Tanon 4500, Shanghai, China).

### Statistical Analysis

GraphPad Prism 8.0 (San Diego, CA, USA) was used for the statistical analysis. Data were expressed as mean ± SD or boxplots (center line indicates the median, the boxes extend to the 25th and 75th percentiles, and the whiskers extend to the minimum and maximum values). Mann–Whitney *U* test (non‐parametric) or two‐tailed unpaired Student's *t*‐test (parametric) was performed for a significant difference between two groups. One‐way analysis of variance (ANOVA) followed by the Tukey test was performed for more than two groups of comparison. ^*^ *p* < 0.05 indicates a significant difference. Other specific statistical analyses were mentioned in each Experimental Section.

## Conflict of Interest

The authors declare no conflict of interest.

## Author Contributions

K.C. and H.G. contributed equally to this work. C.Y., J.C., and R.Q. designed the study. K.C. and H.G. performed all mouse animal experiments and all statistical analyses. Y.Z. and H.X. assisted with animal experiments and experimental parameter determinations. C.Y. obtained funding. K.C. wrote the manuscript, and all authors revised and approved the manuscript.

## Supporting information



Supporting Information

## Data Availability

16S rRNA sequencing data for all samples have been deposited in NCBI and are publicly available as of the date of publication (PRJNA1276697). Any additional information required to reanalyze the data reported in this paper is available from the corresponding authors upon request.
