# Supplementary material for: Disruption of Gut Microbiota‐Mediated De Novo NAD+ Synthesis Contributes to the Development of Polycystic Ovary Syndrome
Source: Adv Sci (Weinh). 2025 Oct 13;12(45):e06497. doi: 10.1002/advs.202506497 (PMC12677664; doi:10.1002/advs.202506497)
Supplement: Supplementary file 1 — Supporting Information [file ADVS-12-e06497-s001.pdf]

Supplemental information for

Disruption of Gut Microbiota-Mediated De Novo NAD$^+$ Synthesis Contributes to the Development of Polycystic Ovary Syndrome

Ke Chen, Huafeng Geng, Yang Zheng, Hongyang Xie, Rui Qin*, Junyang Chen*, Cong Ye*

K. Chen, H. Geng, Y. Zheng, H. Xie, R. Qin, C. Ye
Department of Gynecology
China-Japan Union Hospital of Jilin University
Changchun, Jilin Province, 130033, China.
E-mail: qinrui@jlu.edu.cn; yecong0228@jlu.deu.cn

J. Chen
Department of Anesthesiology
China-Japan Union Hospital of Jilin University
Changchun, Jilin Province 130033, China
E-mail: chenjunyang517@jlu.edu.cn.

The files includes:

Supplementary figures S1-S6

Supplementary figures S1-S6

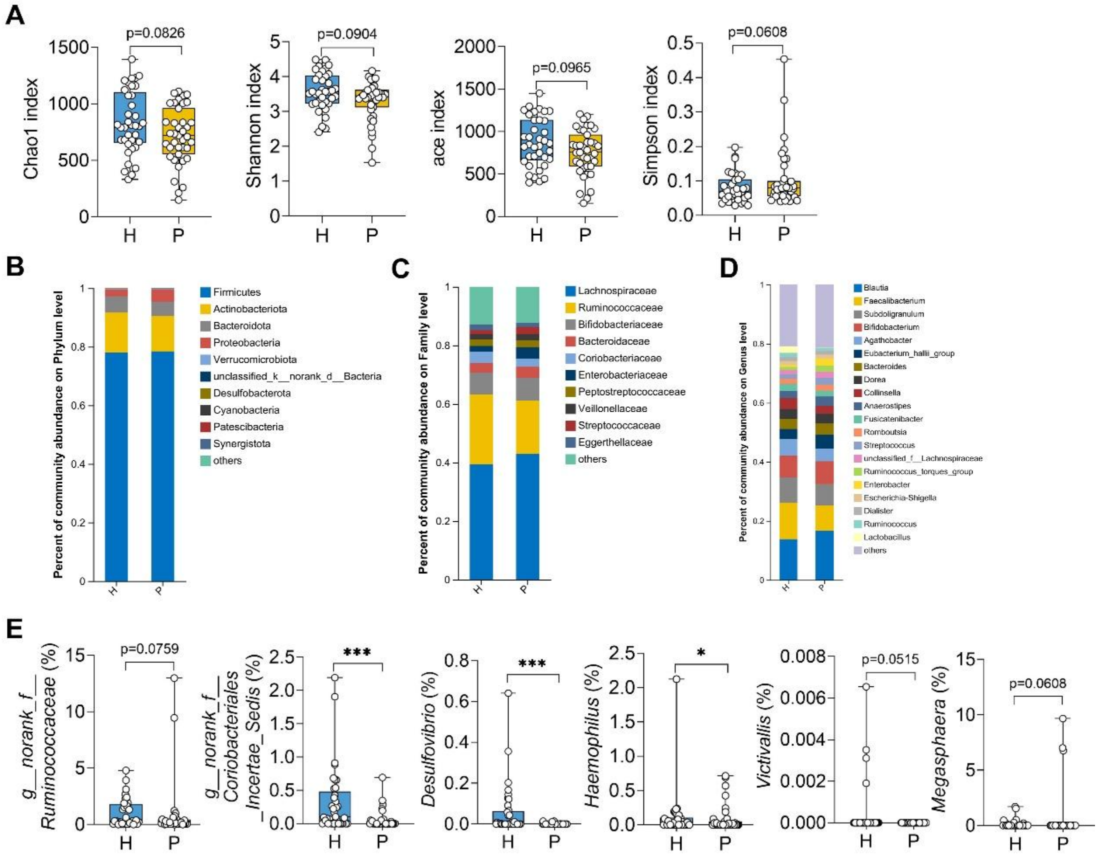

Supplementary Figure. 1 Patients with PCOS displays gut dysbiosis. (A) Alpha diversity indies of the gut microbiota from individuals in the H (Health) and P (PCOS) groups, including Chao1, Shannon, ace and Simpson indices. (B-D) Gut microbial compositions at the phylum (B), family (C) and genus (D) levels. (E) Relative abundances of bacterial taxa enriched in the H and P groups. Data are expressed as boxplot (n=36-39). *$p < 0.05$ and ***$p < 0.001$ by Mann-Whitney *U* test (A and E).

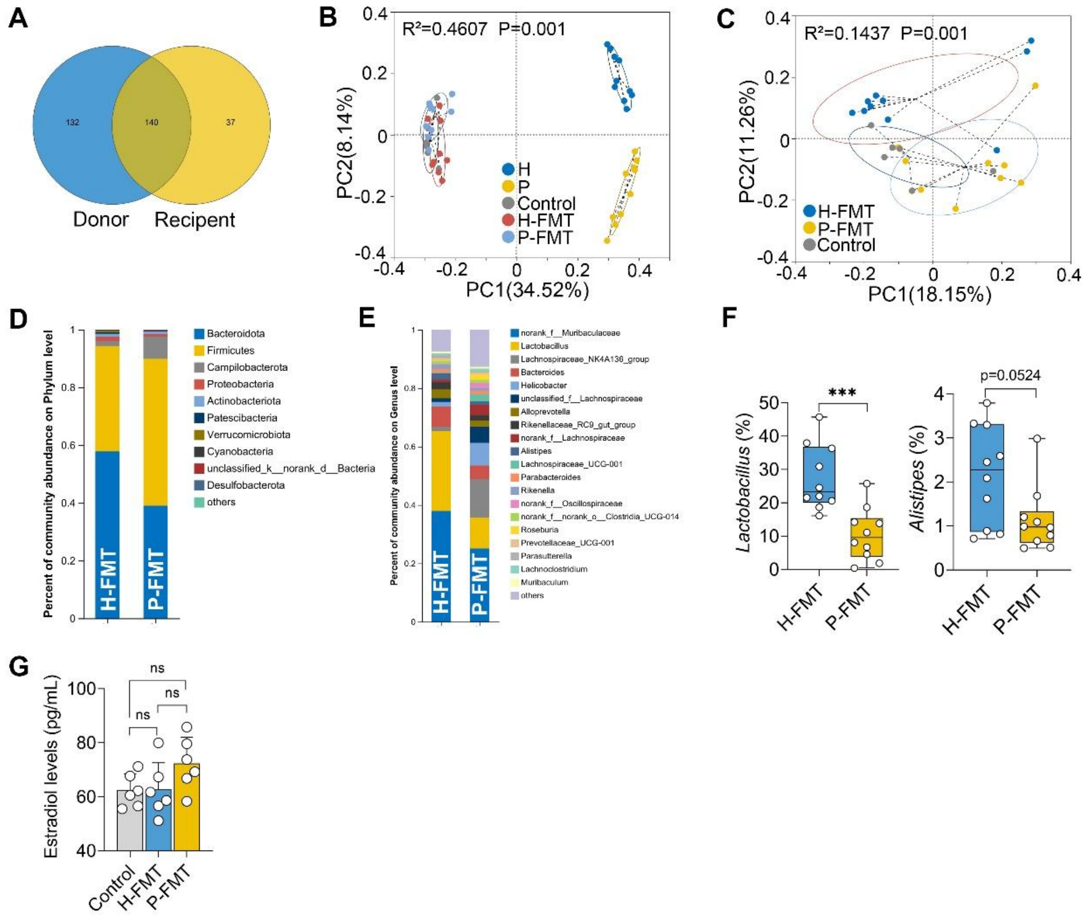

Supplementary Figure. 2 FMT from humans alters the gut microbiota in mice. Mice were orally treated with a cocktail of antibiotics (ABX, 200 mg/kg ampicillin, neomycin and metronidazole, and 100 mg/kg vancomycin) daily for five consecutive days to deplete the original commensal microbes. After replacing ABX by water for one day, these mice were incubated with prepared gut microbial suspension from healthy (H-FMT) or PCOS (M-FMT) patients. (A) Venn diagram showed the number of microbial species in the donors and recipient mice. (B) PCoA score plots showed the gut microbial structure in the donors and recipient mice. (C) PCoA score plots showed that mice from different donors had distinct gut microbial structures. (D and E) Gut microbial compositions at the phylum (D) and genus (E) levels. (F) Relative abundances of bacterial taxa enriched in the H-FMT and P-FMT groups (n=10). (G) Serum estradiol levels from indicated mice (n=6). Data are expressed as boxplot (F and G). ***$p < 0.001$ by Mann-Whitney *U* test (F). ns, no significance.

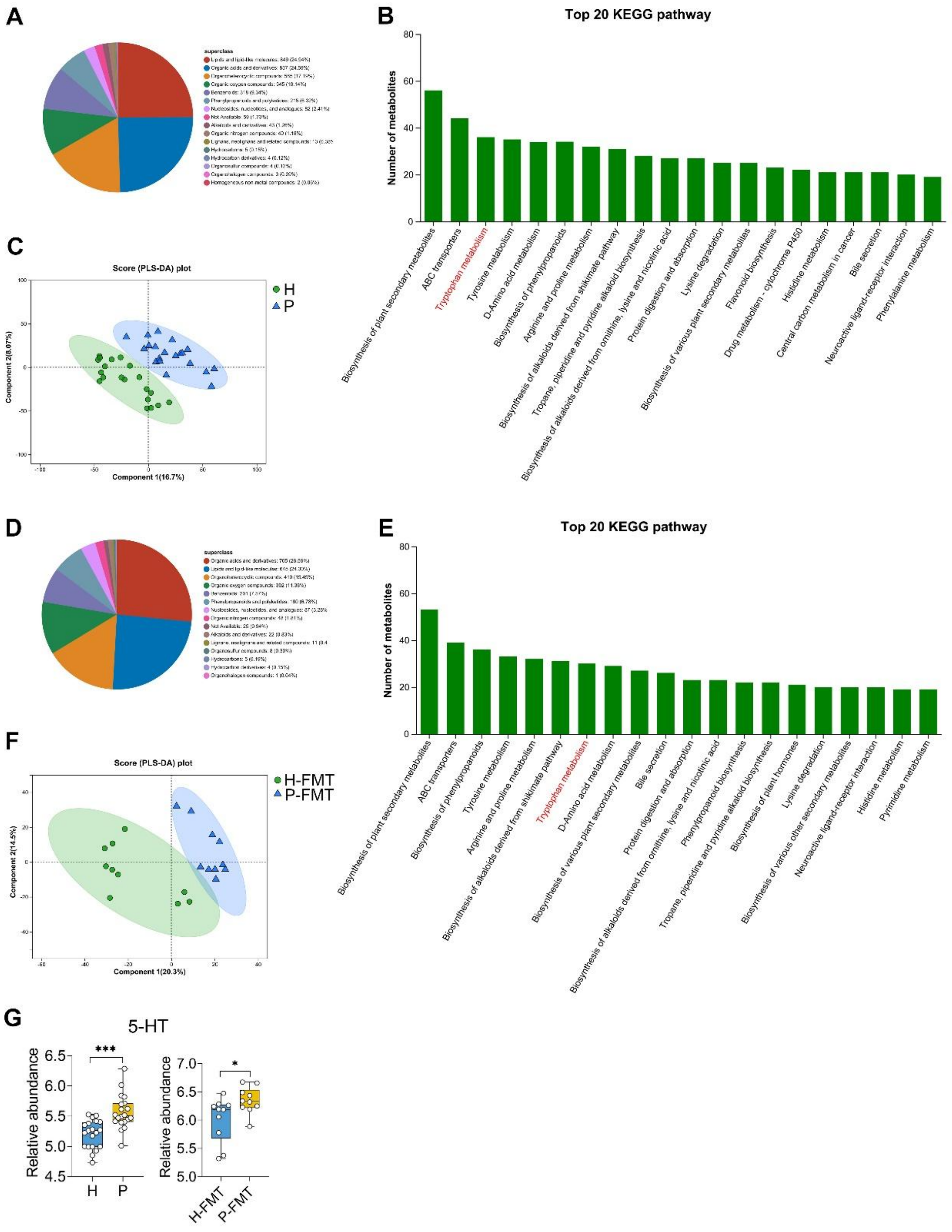

Supplementary Figure. 3 PCOS is associated with gut metabolic changes. (A) Pie chart showed the main fecal metabolite categories from individuals in the H and P groups based on HMDB (Human Motion Database). (B) The top 20 metabolite-enriched KEGG (Kyoto Encyclopedia of Genes and Genomes) pathways in individuals from the H and P groups. (C) PLS-DA score plots of fecal metabolism in the H and P groups (n=20). (D) Pie chart showed the main fecal metabolite categories from individuals in the H-FMT and P-FMT groups based on HMDB. (E) The top 20 metabolite-enriched KEGG pathways of mice in the H-FMT and P-FMT groups. (F) PLS-DA score plots of fecal metabolism in the H-FMT and P-FMT groups (n=10). (G) The relative abundances of 5-HT in humans and recipient mice. *$p < 0.05$ and ***$p < 0.001$ by Mann-Whitney *U* test (G).

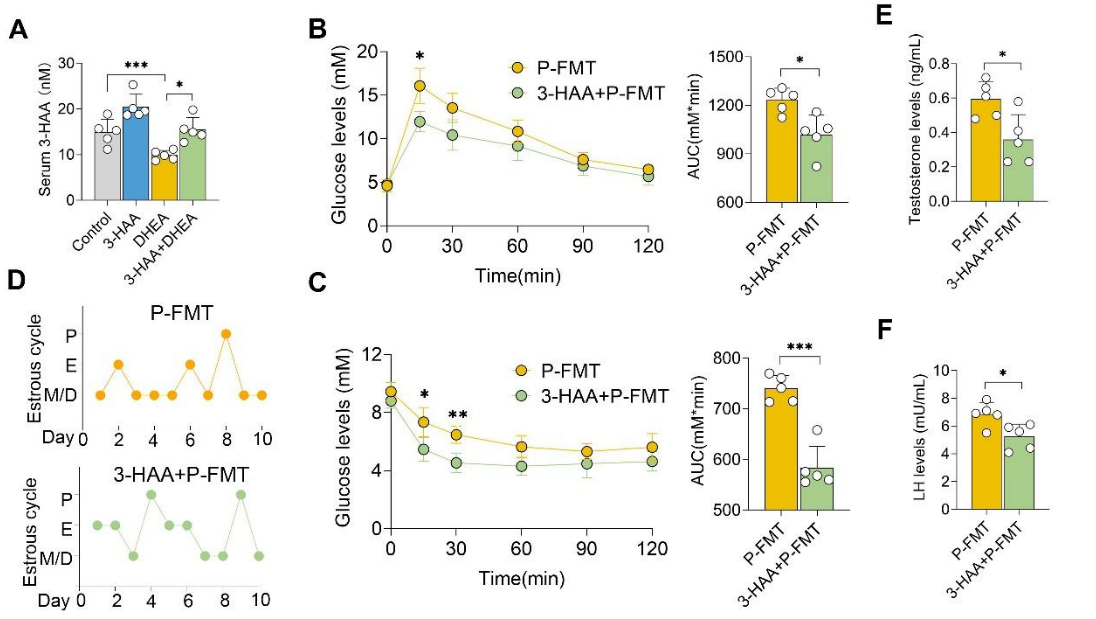

Supplementary Figure. 4 3-HAA alleviates P-FMT-induced PCOS in mice. (A) Serum 3-HAA levels of indicated mice (n=5). B-F, Female mice at prepuberty (21-day-old) were subjected to P-FMT and treated with 3-HAA for 21 days. (B and C) GTT and ITT assays, along with AUC (area under the curve) analysis, in the indicated groups of mice (n=5). (D) Estrous cycle determination based on vaginal smears. P, proestrus; E, estrus; M, metestrus; D, diestrus. (E and F) Serum testosterone and LH levels from indicated mice (n=5). Data are expressed as mean ± SD (n=5). *$p < 0.05$, **$p < 0.01$ and ***$p < 0.001$ by one-way ANOVA followed by Tukey's test (A) and Student's t test (B, C, E and F).

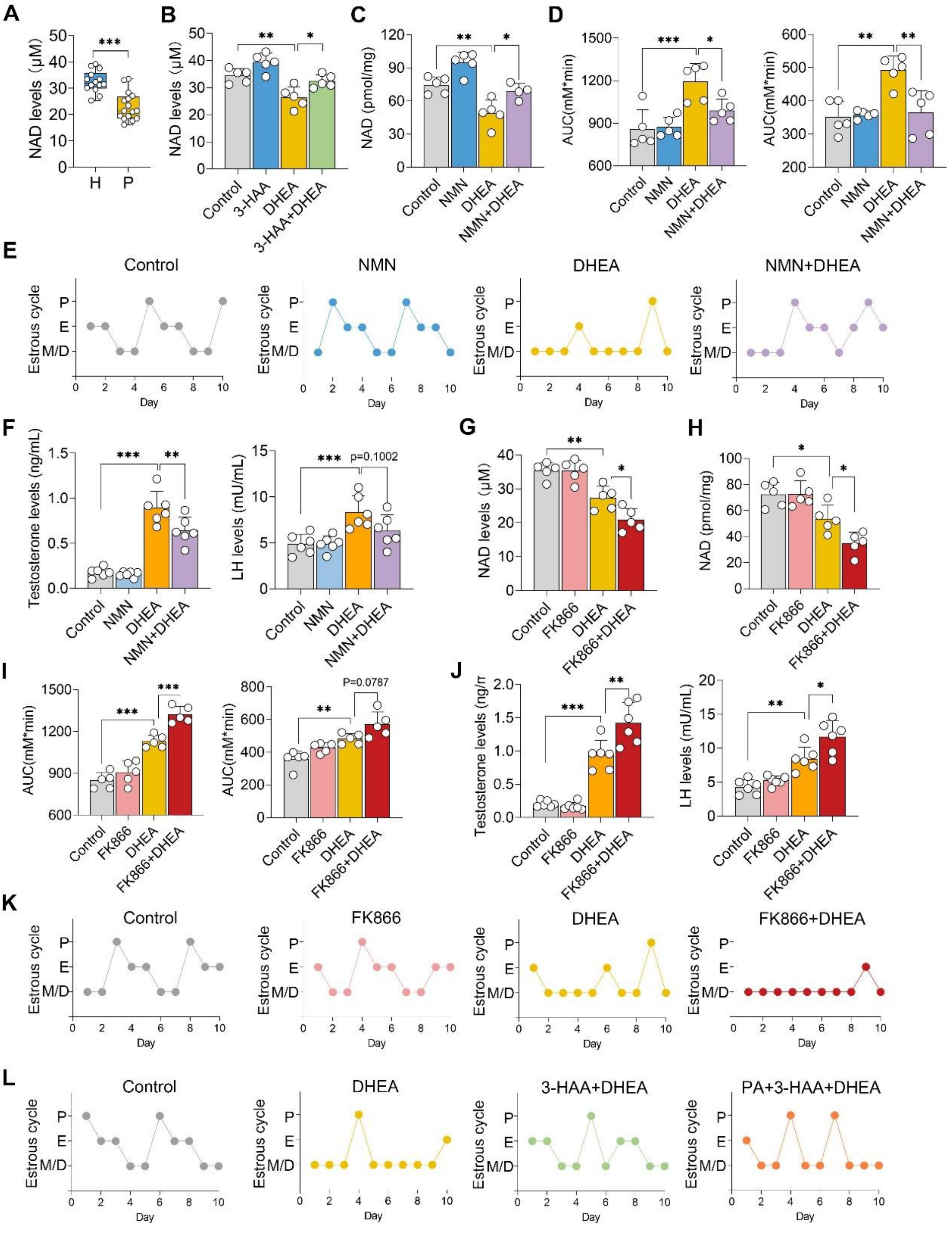

Supplementary Figure. 5 Impaired NAD synthesis contributes to DHEA-induced PCOS in mice. (A and B) Serum NAD^{+} levels in humans (n=14-16) and 3-HAA-treated mice (n=5). C-F, female mice at prepuberal (21-day-old) were intraperitoneally treated with NMN (500 mg/kg) and subcutaneously injected with DHEA (60 mg/kg) daily for 21 days. (C) Serum NAD^{+} levels from indicated mice (n=5). (D) AUC (area under the curve) analysis of GTT and ITT assays in indicated mice (n=5). (E) Estrous cycle determination based on vaginal smears. P, proestrus; E, estrus; M, metestrus; D, diestrus. (F) Serum testosterone and LH levels from indicated mice (n=5). For G-K, female mice at prepuberal (21-day-old) were intraperitoneally treated with FK866 (30 mg/kg) and

subcutaneously injected with DHEA (60 mg/kg) daily for 21 days. (G and H) Serum and ovarian NAD^+ levels in indicated mice (n=5). (I) AUC analysis of GTT and ITT assays in FK866-treted mice (n=5). (J) Serum testosterone and LH levels from indicated mice (n=5). (K) Estrous cycle determination based on vaginal smears. (L) Female mice at prepuberal were intraperitoneally treated with 3-HAA (200 mg/kg) and 200 μL of 100 μM phthalic acid (PA), and subcutaneously injected with DHEA (60 mg/kg) daily for 21 days. Estrous cycle determination based on vaginal smears. Data are expressed as mean ± SD (n=5). *$p < 0.05$, **$p < 0.01$ and ***$p < 0.001$ by one-way ANOVA followed by Tukey's test (A-D and F-J).

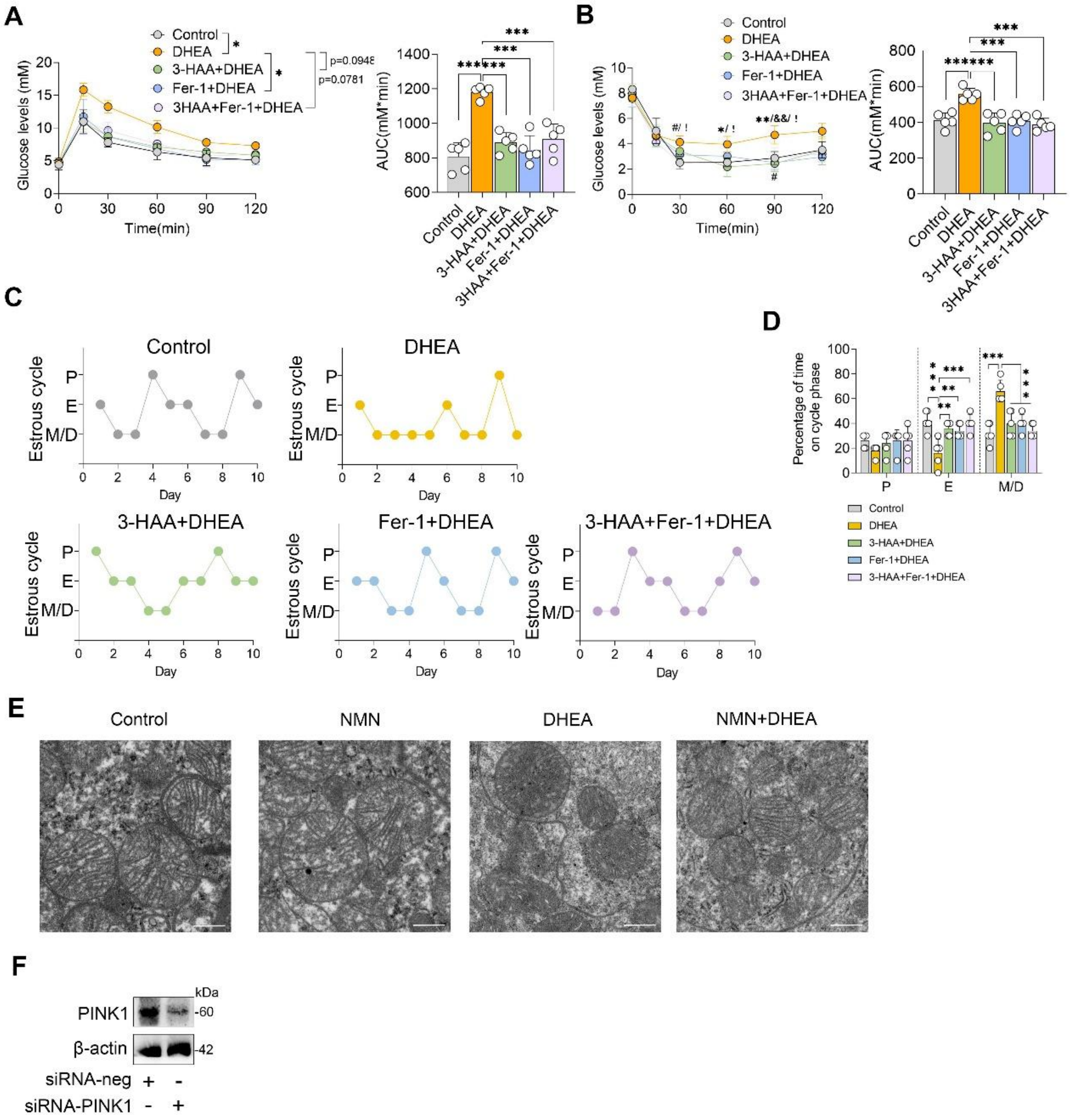

Supplementary Figure. 6 3-HAA alleviates DHEA-induced PCOS by inhibiting ferroptosis. A and B. Prepubertal female mice (21-day-old) were intraperitoneally injected with 3-HAA (200 mg/kg) and Fer-1 (10 mg/kg), and subcutaneously injected with DHEA (60 mg/kg) daily for 21 days. (A) GTT and AUC analysis. (B) ITT and AUC analysis (n=5). *$p < 0.05$ and **$p < 0.01$ compared to the control group; $^{!}p < 0.05$ for 3-HAA+DHEA versus DHEA alone; $^{\&\&}p < 0.01$ for Fer-1+3-HAA versus DHEA alone. (C and D) Estrous cycle determination based on vaginal smears. (E) Mitochondria observation by TEM analysis in NMN-treated cells (scale bar, 500 nm). (F) The expression level of PINK1 was significantly reduced in cells treated with siRNA-PINK1. Data are expressed as mean ± SD (n=5). ***$p < 0.001$ by two-way ANOVA (A and B) and one-way ANOVA followed by Tukey's test (A, B and D).
